# Involving Patients and Clinicians in the Design of Wireframes for Cancer Medicines Electronic Patient Reported Outcome Measures in Clinical Care: Mixed Methods Study

**DOI:** 10.2196/48296

**Published:** 2023-12-21

**Authors:** Emma Dunlop, Aimee Ferguson, Tanja Mueller, Kelly Baillie, Jennifer Laskey, Julie Clarke, Amanj Kurdi, Ann Wales, Thomas Connolly, Marion Bennie

**Affiliations:** 1 Strathclyde Institute of Pharmacy & Biomedical Sciences University of Strathclyde Glasgow United Kingdom; 2 NHS Lanarkshire Wishaw United Kingdom; 3 NHS Greater Glasgow & Clyde Glasgow United Kingdom; 4 Department of Pharmacology, College of Pharmacy Hawler Medical University Erbil Iraq; 5 Department of Clinical Pharmacy, College of Pharmacy Al-Kitab University Kirkuk Iraq; 6 NHS Healthcare Improvement Scotland Glasgow United Kingdom; 7 DS Partnership Glasgow United Kingdom

**Keywords:** cancer, clinicians, mHealth, mixed methods study, patient reported outcome measures, patients, technology acceptance model

## Abstract

**Background:**

Cancer treatment is a key component of health care systems, and the increasing number of cancer medicines is expanding the treatment landscape. However, evidence of the impact on patients has been focused more on chemotherapy toxicity and symptom control and less on the effect of cancer medicines more broadly on patients’ lives. Evolving electronic patient-reported outcome measures (ePROMs) presents the opportunity to secure early engagement of patients and clinicians in shaping the collection of quality-of-life metrics and presenting these data to better support the patient-clinician decision-making process.

**Objective:**

The aim of this study was to obtain initial feedback from patients and clinicians on the wireframes of a digital solution (patient app and clinician dashboard) for the collection and use of cancer medicines ePROMs.

**Methods:**

We adopted a 2-stage, mixed methods approach. Stage 1 (March to June 2019) consisted of interviews and focus groups with cancer clinicians and patients with cancer to explore the face validity of the wireframes, informed by the technology acceptance model constructs (perceived ease of use, perceived usefulness, and behavioral intention to use). In stage 2 (October 2019 to February 2020), the revised wireframes were assessed through web-based, adapted technology acceptance model questionnaires. Qualitative data (stage 1) underwent a framework analysis, and descriptive statistics were performed on quantitative data (stage 2). Clinicians and patients with cancer were recruited from NHS Greater Glasgow & Clyde, the largest health board in Scotland.

**Results:**

A total of 14 clinicians and 19 patients participated in a combination of stage 1 interviews and focus groups. Clinicians and patients indicated that the wireframes of a patient app and clinician dashboard for the collection of cancer medicines ePROMs would be easy to use and could focus discussions, and they would be receptive to using such tools in the future. In stage 1, clinicians raised the potential impact on workload, and both groups identified the need for adequate IT skills to use each technology. Changes to the wireframes were made, and in stage 2, clinicians (n=8) and patients (n=16) indicated it was “quite likely” that the technologies would be easy to use and they would be “quite likely” to use them in the future. Notably, clinicians indicated that they would use the dashboard to enable treatment decisions “with around half” of their patients.

**Conclusions:**

This study emphasizes the importance of consulting both patients and clinicians in the design of digital solutions. The wireframes were perceived positively by patients and clinicians who were willing to use such technologies if available in the future as part of routine care. However, challenges were raised, and some differences were identified between participant groups, which warrant further research.

## Introduction

There are approximately 393,000 cancer diagnoses per year in the United Kingdom [1]. The number of cancer medicines is increasing, resulting in a rapidly expanding treatment landscape and new treatment pathways for patients [2]. Evidence from a review suggests that overall survival (OS) is increasing for many cancers due to the availability of new cancer medicines [3]. However, evidence of the impact on patients’ quality of life (QoL) has focused principally on chemotherapy toxicity and symptom management [4-8]. Less is known about the impact of cancer medicines more broadly on patients’ lives; this is often an important consideration in the patient-clinician decision-making process.

The use of patient-reported outcome measures (PROMs) as part of clinical care has been shown to improve QoL and OS [9], support self-management and patient satisfaction with care [10,11], and facilitate communication between patients and clinicians [6,12,13]. PROMs can also assist in identifying patients’ supportive care needs and provide an opportunity to address sensitive issues or unexpected concerns [6,11,14,15]. Building on broader technological advancements, there is an increasing focus on and support for using digital tools to capture electronic PROMS (ePROMs) and their integration into routine clinical cancer care systems [9,16-20].

Effectively engaging with stakeholders, clinicians, and patients in particular is important to create usable, relevant, and effective ePROMs solutions that can support care [21-26]. However, evidence indicates that patients are infrequently involved in the design of health apps [27,28], often less so than clinicians [26,29], frequently only at the validation or piloting stages, and not as early in the process as clinicians [26,30]. Furthermore, a review of reviews found little evidence of patients being involved in the design of PROMs processes [23].

In Scotland, there is government support for involving citizens and end users in the design of tools and technologies as part of digital health and care provision, with a view to creating tools for successful adoption [31]. The 2023 Scottish Cancer Strategy [32,33] has committed to assessing the potential of ePROMs to assist in the delivery of cancer services, enabled in part through the Scottish government–funded Cancer Medicines Outcomes Programme (CMOP) to explore the capabilities of collecting cancer medicines ePROMs as part of routine care [31,33].

The aim of this study was to obtain initial feedback from patients and clinicians on the wireframes of a digital solution (patient app and clinician dashboard) for the collection and use of cancer medicines ePROMs.

## Methods

### Overview

We adopted a 2-stage, mixed methods approach. First, interviews and focus groups with cancer clinicians and patients with cancer to test wireframes of a clinician dashboard and a patient app, respectively, for face validity (stage 1) were conducted. Second, based on refinements informed by the first stage, a review of the revised wireframes was conducted with clinicians and patients through web-based questionnaires (stage 2).

### Research Material Development

The technology acceptance model (TAM) [34,35], a commonly used framework for studying health technology acceptance behavior [36], underpinned the design of the study. According to the TAM, an individual’s behavioral intention to use a new technology is determined by their attitude toward the use of technology, which is influenced by perceived ease of use (the user’s “subjective probability” that using the technology would enhance their performance) and perceived usefulness (the degree to which an individual believes that a technology would be “free of effort”) [35]. The TAM was used as a framework upon which the interview schedules and questionnaires described were based.

All study materials were validated by members of the extended project team to ensure the appropriateness and clarity of the questions and the language used.

### App and Dashboard Wireframe Development

App and dashboard wireframes (final versions in Multimedia Appendix 1) were designed by Tactuum [37], a digital solutions company, in collaboration with the CMOP Research Team and were in jpeg format. The content of the wireframes was based on 2 PROMs tools that aligned with patient and clinician QoL priorities identified in an earlier study [38]. These were the National Comprehensive Cancer Network (NCCN) Distress Thermometer and Problems List [39,40] and the EQ-5D-5L [41]. A description of how these 2 tools were identified can be found in Multimedia Appendix 2 [38,39,41].

### Participant Information Sheets, Consent Forms, and Demographics

#### Overview

Participant information sheets (PIS) for each participant group at each study stage were developed and provided to participants before the interviews and focus groups commenced. Informed consent was obtained through consent forms, which were developed using the University of Strathclyde standard templates. Demographics sheets contained common questions across both participant groups and at both stages (eg, age and gender) and specific questions on job role and clinical area of expertise for clinicians, time since diagnosis, and frequency of clinic visits for patients.

#### Stage 1: Interview and Focus Group Schedules

Semistructured interview and focus group schedules were developed for clinicians and patients, respectively, using the constructs of the TAM to explore the perceived ease of use, usefulness, and intention to use the app or dashboard. For context, the clinician interview schedule (Multimedia Appendix 3) also included questions on how clinicians operate their clinics and how QoL is currently recorded and discussed with patients, if at all. The patient focus group schedule (Multimedia Appendix 4) also included questions on how patients record and discuss their QoL with their clinicians, if at all, and covered their general use of mobile technologies and apps for context.

#### Stage 2: Web-Based Questionnaires

Based on the findings from stage 1, changes were made to the app and dashboard wireframes, and these refined versions were embedded within web-based questionnaires (Multimedia Appendices 5 and 6) as jpeg images. The web-based questionnaires were developed by adapting the TAM questionnaire to our particular study (eg, adding the words “CMOP PROMs app” into question wording) [34]. Most questions required Likert-type responses; 1 question required a response using a sliding scale of 0-100; and 1 question used multiple category choices. The web-based questionnaire was hosted on Qualtrics (Qualtrics LLC).

### Participants

#### Overview

Participants were identified through the CMOP network established in NHS Greater Glasgow & Clyde (NHS GGC), the largest health board in Scotland, to conduct clinical studies in specific cancer areas (ie, prostate, gynecological, and melanoma).

Initially, clinicians working in prostate cancer, gynecological cancers, and melanoma in NHS GGC, aligned to CMOP, were identified. Additional participants were recruited through snowball sampling, where the identified clinicians were asked to forward details of the study to their clinical colleagues.

In stage 1, only patients attending prostate cancer support groups known to the CMOP network in NHS GGC were recruited. In stage 2, patient recruitment was extended to patients attending prostate cancer, gynecological cancers, and melanoma clinics from 2 NHS GGC hospitals. Patients were recruited by asking clinicians from stage 1 to distribute copies of a stage 2 PIS to patients in clinic, which contained a URL link to the stage 2 patient web-based questionnaire. Some clinicians also provided copies of the patient PIS to colleagues for further distribution while covering their clinics.

#### Data Collection and Analysis

**Figure 1:**
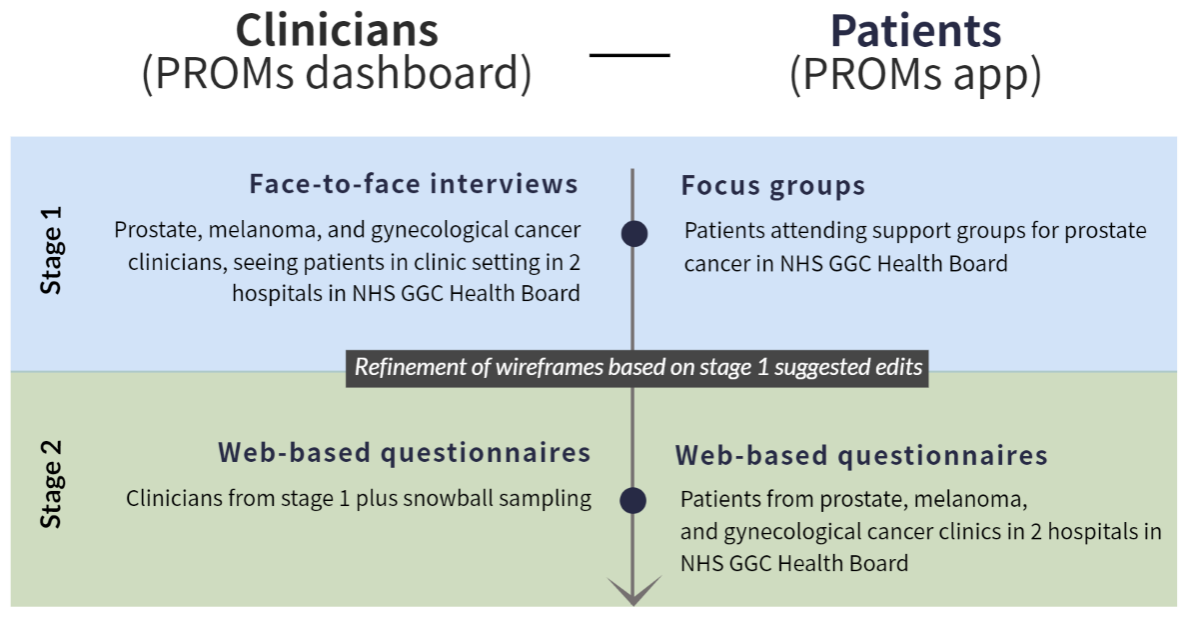
Data collection methods. NHS GGC: NHS Greater Glasgow & Clyde; PROMs: patient-reported outcome measures.

Figure 1 provides an overview of the data collection methods.

#### Stage 1

Clinicians participated in interviews between March and April 2019. The PROMs dashboard wireframes were presented using a tablet device during the interview. Patients participated in focus groups between May and June 2019, where app wireframes were shown either on a tablet or a projector screen.

All interviews and focus groups were audio recorded and transcribed using an intelligent verbatim approach, and all transcriptions were validated. The data were analyzed thematically by 1 researcher in NVivo (version 12; QSR International) using the 3 constructs of the TAM as high-level themes: perceived usefulness, perceived ease of use, and behavioral intention to use. Emergent themes and subthemes below these were derived. All coding was validated by a second researcher.

Suggested edits to the wireframes were identified from the interviews and focus groups. Edits that could be made to a jpeg format were executed by the research team and embedded within the web-based questionnaires for stage 2. However, some suggested changes would have required significant resources to develop additional wireframes or a working prototype and were considered by the research team not to impact the intended general purpose and function of the app or dashboard. Therefore, these edits were not made on the wireframes for stage 2 (see the *Results* section).

#### Stage 2

The clinician and patient web-based questionnaires are reported using the Checklist for Reporting Results of Internet E-Surveys (CHERRIES) checklist in Multimedia Appendix 7 [42]. In summary, clinicians from stage 1 were emailed a link to the web-based questionnaire in October 2019. Reminder emails were sent out to those who had not yet completed the questionnaire within a month of receiving the initial invitation to participate. Responses were collected until December 2019.

Patients were invited to complete the web-based questionnaire between November 2019 and February 2020.

All questionnaire data was extracted from Qualtrics and imported into Microsoft Excel (Microsoft Corporation). For the quantitative data, basic descriptive analyses (median and IQR) were performed for each individual question, where appropriate. Some values were reverse coded depending on the response scale for consistency in reporting. Overall medians and IQRs based on the TAM constructs were calculated for Likert-type responses to understand, more generally, perceptions on how easy to use, useful, and likely to be used the respective technologies are. Free text data were summarized narratively.

### Ethical Considerations

This study was granted ethical approval by the University of Strathclyde Ethics Committee (UEC19/17). Participants provided informed consent at each stage of the study after reading the relevant PIS and signing a consent form based on the University of Strathclyde’s standard template. The PIS and the consent forms assured participants that their participation was voluntary, that their data would be anonymized, and that they would remain anonymous in the reporting of the research. Participants were informed about their freedom to refuse**.** Participants were not compensated for taking part in the study.

## Results

### Demographics

Table 1 provides the participant demographics.

**Table 1:** Clinician and patient demographics stages 1 and 2.

Demographics				Clinicians		Patients	
				Stage 1 (n=14)	Stage 2 (n=8)	Stage 1 (n=19)	Stage 2 (n=16)
Age (years), median (IQR)^a^				50 (41-52)	48.9 (46.3-52.5)	67 (64-70)	67.5 (56.3-75.3)
**Gender, n (%)**							
	Woman			12 (86)	6 (75)	0 (0)	4 (25)
	Man			2 (14)	2 (25)	19 (100)	12 (75)
**Cancer type^b^, n (%)**							
			Urology or prostate	6 (43)	4 (50)	19 (100)	3 (19)
			Melanoma	4 (29)	1 (13)	N/A^c^	11 (69)
			Gynecology	6 (43)	4 (50)	N/A	1 (6)
			Other^d^	3 (21)	2 (25)	N/A	1 (6)
**Job role, n (%)**							
			Consultant oncologist	8 (57)	5 (63)	N/A	N/A
			Clinical nurse specialist	3 (21)	2 (25)	N/A	N/A
			Pharmacist	1 (7)	1 (13)	N/A	N/A
			Other^e^	2 (14)	0 (0)	N/A	N/A
Time in job role (years), median (IQR)				11 (4.25-13)	9 (4.8-13.8)	N/A	N/A
**Time since diagnosis, n (%)**							
			Less than 1 year	N/A	N/A	5 (26)	5 (31)
			Between 1-5 years	N/A	N/A	10 (53)	7 (44)
			More than 5 years	N/A	N/A	4 (21)	4 (25)
**Clinic visit frequency, n (%)**							
		Every 3-4 weeks		N/A	N/A	3 (16)	12 (75)
		Every 3 months		N/A	N/A	9 (47)	3 (19)
		Every 6 months		N/A	N/A	2 (11)	1 (6)
		Once a year		N/A	N/A	1 (5)	0 (0)
		Not sure or not applicable		N/A	N/A	3 (16)	0 (0)

^a^A total of 2 clinicians did not provide a response.

^b^The number of clinicians working in each cancer area. Some clinicians were working in more than 1 clinical area.

^c^Not applicable.

^d^Clinicians worked in other cancer areas, including thyroid, endocrine, hepato-pancreato-biliary, and gastrointestinal cancers. A total of 1 patient did not define what cancer diagnosis they had received.

^e^A total of 2 participants who selected “other” identified as doctors in training.

### Stage 1: Interviews and Focus Groups

#### Clinician Interviews on Dashboard

##### Perceived Ease of Use

Clinicians reported that the dashboard looked easy to operate and looked user-friendly; however, some said they would have to go to some effort to remember to use it.

when you’ve got so many patients and you’re so busy trying to remember to do things in a routine way can be difficult.

Oncologist, melanoma

Some clinicians expressed concerns related to patients having sufficient IT literacy to input data through an app, especially those who were older.

I think for younger patients they’re ok because they’re technical things and the elder patient’s problem is that it’s quite technical.

Doctor in training, melanoma

However, some clinicians disagreed.

Patients surprise you, it would be easy to make assumptions that somebody a bit older doesn’t like to use computers. So many older people have phones who know what they’re doing.

Clinical nurse specialist (CNS), neuroendocrine and thyroid

##### Perceived Usefulness

Despite fears that the dashboard would constrain time in the clinic, some clinicians said it would enable them to use their time better to focus on issues and discuss them more fully.

It might remind me to come back to a question that I haven’t asked, because sometimes patients leave and I have thought “oh gosh I’d written that down and didn’t bring it up today”.

CNS, urology

Some worried that the dashboard could add workload and time pressures, especially if patient responses raised additional issues, “because once you’ve asked it you can’t really ignore it” (CNS, melanoma). However, the potential benefits of replacing currently used, paper-based notes on patients’ QoL and lessening this risk of duplication of efforts were mentioned.

Clinicians generally said using the dashboard would facilitate shared decision-making.

It will help when we have conversations about whether it’s right to continue treatment...Having that visual evidence of how they’re feeling would be helpful.

Oncologist, gynecology

Some clinicians said that the dashboard would at least provide supporting evidence for decisions already being made: “I don’t think that would influence the decision...but [the dashboard information] might help” (CNS, urology). This could be useful with new or less familiar patients, given the lack of previous knowledge of their concerns.

You see somebody who’s sick and you've never met them before, you say, well, is this new, have they always been like that?

Oncologist, urology

The dashboard was perceived as potentially useful when used alongside standard protocols.

I have to rely on clinical markers from a safety point of view...I would make a decision on what we would do based on that whole picture.

Pharmacist, Gynecology

Aggregate data within the dashboard on all clinic patients was seen as useful to some, potentially being more realistic and beneficial than clinical trial data: “So when we’re telling patients this is the likelihood you’ll get this issue...This is giving them a more realistic view” (CNS, melanoma). Usefulness would also depend on digital integration, as clinicians reported already using various IT systems.

it’s another system to go in and look at...because although you wouldn’t necessarily be going into [System 1 and System 2] for a whole clinic set, all of those are open on your desktop...you’re not individually going in every time.

Oncologist, urology and gynecology

Finally, concerns were raised around usefulness as patients vary, as do their supportive care needs, by cancer type, treatment, and priorities.

I do such a wide range of things...it depends whether they’re palliative...whether or not I’m trying to treat them with the intent to cure them...really depends on the patient, how much you’re using that information.

Oncologist, urology and gynecology

##### Behavioral Intention

Overall, most clinicians said they would intend to use a dashboard for viewing PROMs data.

You don’t have time necessarily to deal with 18 different things...and the main thing that was causing them bother was their breathing you could say, ‘okay, today we're going to talk about getting this sorted’...that’s much more realistic.

Oncologist, gynecology

One of the risks identified was the potential disruption to normal clinical flow while getting used to using a new tool. Clinicians said they were likely to use the dashboard with some patients, perhaps certain groups depending on their treatments and needs: “I think the abiraterone and enzalutamide patients are a sort of good group.” (CNS, urology).

#### Patient Focus Groups on App

##### Perceived Ease of Use

Patients said the app wireframes looked easy to use.

I’d be quite happy with having something that’s as accessible as that to operate, I think it’ll get easier...when you start a new app it’s new to you.

Focus group 3, participant 1

Some patients mentioned potentially needing training to use an app like this: “You just need to give us a wee bit of training on it and show us how it works” (focus group 1, participant 1).

##### Perceived Usefulness

Patients liked that the app would help them focus on areas to discuss in the clinic: “It would force you to think about it, and then list the questions you want to ask. It would focus your mind” (focus group 2, participant 3).

Patients said the app would be useful for tracking their progress during treatment: “It would be nice to see some progress because it’s a very dark place initially” (focus group 1, participant 1). Patients also recognized that the data collected could benefit others.

There’s got to be something that shows what you went through, that there is hope out there. That’s got to be the first thing that’s fed into people’s heads.

Focus group 1, participant 7

##### Behavioral Intention

Patients said they would use the app and that it could also act as a personal diary.

It’s like an online diary and you’re recording where you are at that moment in time and you can see the journey, your progression.

Focus group 3, participant 1

Some preferred the idea of a website rather than an app due to issues with signal on mobile devices.

very often the signal can be poor but I have more faith in a website on a laptop, [that] would be my first choice.

Focus group 2, participant 6

A website’s more efficient than an app.

Focus group 2, participant 2

#### Proposed Dashboard and App Wireframe Changes

Multimedia Appendix 8 contains details on all suggested changes.

Participants made suggested changes to the wireframes that impacted the ease of use and usefulness of the technologies. For the clinician dashboard, text and visual elements were resized, performance status was added, and a clearer indication that a symptom or side effect was new (not just if it was present) was added.

Changes to the patient app were mostly around accessibility and ease of use (eg, “Yes” or “No” buttons rather than toggles for responding to questions, an increase in text size, fewer questions per page to reduce the need for scrolling, “Next” and “Back” buttons, and a variety of sign-in options), and changing the 0-100 scale to a scale of 1-10.

### Stage 2: Web-Based Questionnaire

#### Overview

Multimedia Appendix 9 provides the overall median (IQR) for perceived ease of use, perceived usefulness, and behavioral intention for each participant group, in addition to results for each individual question.

Clinicians and patients reported that it was “quite likely” that the technologies would be easy to use and would be “quite likely” to be used in the future. However, the median score for perceived usefulness was higher for patients than clinicians, “quite likely” versus “neither likely nor unlikely,” respectively. What follows is a descriptive summary of responses to the stage 2 questionnaires.

#### Clinician Dashboard

Overall, clinicians thought the dashboard was likely easy and flexible to use, and they would likely find it easy to learn how to use it. Although clinicians considered PROMs highly important, they reported that PROMs were only slightly relevant to their decision-making; nevertheless, they reported that they would likely use the dashboard on a regular basis. Clinicians were asked to score 0-100 on what the chances were that they would use the dashboard in the future, and the median response was 70 out of 100. Clinicians reported they would use the tool to enable treatment decisions with “around half” of their patients.

#### Patient App

In general, patients thought the app would be both easy and flexible to use, and they would likely find learning how to use it easy. Patients perceived the app to likely enable them to communicate how their treatment impacts their QoL, and they thought that using the app would likely enhance the effectiveness of their treatment decision-making. Patients reported that how their treatment impacts their QoL is highly important and highly relevant; they thought that using the app would likely improve their QoL. Patients were asked to score from 0 to 100 on what the chances were that they would use an app like this in the future, and the median response was 75 out of 100, with patients reporting that they would use it “before most” or “every clinic appointment.”

## Discussion

### Principal Findings

The principal results of this study showed that clinicians and patients indicated that the wireframes of a patient app and clinician dashboard for the collection of cancer medicines PROMs would be easy to use and could focus discussions, and they would be receptive to using such tools in the future. In stage 1, clinicians raised the potential impact on clinician workload, and both groups identified the need for adequate IT skills to use the app and dashboard. Changes to the wireframes were made, and in stage 2, clinicians and patients indicated it was “quite likely” that the technologies would be easy to use and they would be “quite likely” to use them in the future. Patients and clinicians also scored similarly when asked what chance there was of them using the app or dashboard in the wireframes (median 75 out of 100 and 70 out of 100 chance, respectively). Notably, clinicians indicated that they would use the dashboard to enable treatment decisions with “around half” of their patients.

### Comparison With Other Work

Previously, clinicians have reported that they would find an ePROMs solution easy to use and useful [43], as seen in this study. In this study, clinicians expressed worries around additional time, workload, and challenges around data integration with existing IT systems, also seen previously [13,18,43,44]. Perceived benefits highlighted in this study, such as being able to identify specific symptoms, side effects, and supportive care needs of patients, have also been reported elsewhere [43]. Similarly, patients in this study reported that using a digital solution for collecting ePROMs would be acceptable as part of routine care. Previous evidence has shown that collecting PROMs in routine care could help bring key issues to the fore [15-17,45-48]. Furthermore, in this study, ePROMs were seen as potentially valuable in terms of contributing to the wider evidence base, as reported previously [47]. The importance of PROMs for identifying patients’ supportive care needs was observed both in this study and in the literature [6,11,14].

This study also identified some interesting contrasting views both within the clinician group and between patients and clinicians. First, clinicians reported that the dashboard would not necessarily improve the effectiveness or productivity of treatment decision-making, nor would it make their job easier, despite saying that the dashboard would help support or facilitate shared decision-making in stage 1. Similarly, in the literature, PROMs are generally seen as useful for clinical decision-making, although they may not always directly impact the decisions made [13,14,47]. Our findings indicate that clinicians may possibly see PROMs as supporting decisions already being made, as opposed to changing the course of decisions entirely. Nevertheless, this did not appear to impact the perceived usefulness of the clinician dashboard. Second, patients’ age was suggested as a possible barrier to use by some clinicians, as articulated in previous studies [17,20,46,48]. However, despite suggesting changes relating to the accessibility of the app, other clinicians as well as patients (median age of 67 years) indicated they would use the technology and did not report age as a barrier.

### Strengths and Limitations

The main strengths of the study were the mixed methods approach and the involvement of patients and clinicians in the wireframe design. The TAM has been used previously to explore patient and clinician views on telehealth or web-based patient monitoring technologies in both qualitative and quantitative studies [49-51]. The ambition that the app and dashboard wireframes had a broad cancer medicine focus—as opposed to specific cancers or treatments—fills a recognized gap in the current evidence base. As illustrated in our results, the changes between stages 1 and 2 wireframes were not major, and the wireframes at both stages were generally acceptable to participants, which provides a degree of confidence in our findings.

In terms of limitations, the sample size was small as we used existing CMOP relationships with clinicians and patient groups for recruitment. We acknowledge that stage 1 patient recruitment only included men with prostate cancer, and consequently, in stage 2, we extended the types of cancer represented to gynecological and melanoma. Given the intention was to obtain initial feedback to inform the design of wireframes, we considered 3 cancer types acceptable for this level of development. Due to the use of snowballing as part of the recruitment strategy, we were unable to calculate a participation rate for the stage 2 questionnaires; however, all clinicians who started the stage 2 questionnaire completed it (completion rate=100%). The stage 1 interview and focus group schedules and stage 2 questionnaires were not piloted but underwent validation by members of the research team, with experience in research methods and cancer patient care, to ensure they were appropriate and clear. Finally, not all changes suggested by clinicians and patients could be made to the existing jpeg wireframes for stage 2 evaluation, as some would have required the development of a working prototype, which was beyond the scope of the study.

### Conclusions

There is a rapidly growing interest in app-based ePROMs solutions and how they could improve clinical practice. This study emphasizes the importance of consulting both patients and clinicians in the design of digital solutions and highlights some perceived challenges in ePROMs use by clinicians. These challenges need to be brought to the fore in any future design or implementation of ePROMs in routine cancer care.

Future research should focus more on the potential conflict between how useful ePROMs are perceived to be and how this may or may not influence treatment decision-making. Although clinicians reported an intention to use ePROMs, the value that ePROMs may have in treatment decisions needs further exploration. Future research should explore the potential discrepancies between clinician and patient perceptions of patient eHealth literacy. Although the literature indicates that older patients may engage less with digital solutions, there is some evidence to support the contrary in this study. Further exploration should aim to resolve any misplaced age-related bias around the uptake of technology as part of clinical care.

Building on these findings in line with Scotland’s vision for a strategic, coordinated approach to the adoption of ePROMs [32,33], the Scottish Cancer PROMs Advisory Group and Forum have been established. The aim of this group is to guide clinical practice, research, and policies relevant to the use of digital PROMs for patients with cancer in Scotland and provide a collaborative space for discussion and shared learning across multiple stakeholders (including patients and members of the public) to achieve our national ambition: to work toward a cohesive approach to providing cancer care across Scotland and beyond.

## Appendix

Multimedia Appendix 1 Final app and dashboard wireframes.

Multimedia Appendix 2 Description of patient-reported outcome measures (PROMs) tool selection process.

Multimedia Appendix 3 Stage 1 clinician interview schedule.

Multimedia Appendix 4 Stage 1 patient focus group schedule.

Multimedia Appendix 5 Stage 2 clinician questionnaire.

Multimedia Appendix 6 Stage 2 patient app questionnaire.

Multimedia Appendix 7 Stage 2 Clinician and Patient web-based questionnaire: Checklist for Reporting Results of Internet E-Surveys (CHERRIES) checklists.

Multimedia Appendix 8 Summary of changes to clinician dashboard and patient app from stage 1.

Multimedia Appendix 9 Stage 2 clinician and patient median and IQR responses.

## Abbreviations

CHERRIES: Checklist for Reporting Results of Internet E-Surveys
CMOP: Cancer Medicines Outcomes Programme
CNS: clinical nurse specialist
ePROMs: electronic patient-reported outcome measures
NCCN: National Comprehensive Cancer Network
NHS GGC: NHS Greater Glasgow & Clyde
OS: overall survival
PIS: participant information sheets
PROM: patient-reported outcome measure
QoL: quality of life
TAM: technology acceptance model

## Data Availability

All data generated or analyzed during this study are included in this published article and its multimedia appendices.
