# Supplementary material for: Involving Patients and Clinicians in the Design of Wireframes for Cancer Medicines Electronic Patient Reported Outcome Measures in Clinical Care: Mixed Methods Study
Source: JMIR Form Res. 2023 Dec 21;7:e48296. doi: 10.2196/48296 (PMC10767627; doi:10.2196/48296)

# Multimedia Appendix 1: Final App and Dashboard Wireframes (at Stage 2)

This is a Multimedia Appendix 1 for a full manuscript published in JMIR Formative Research. For full copyright and citation information see “Involving Patients and Clinicians in the Design of Wireframes for Cancer Medicines Electronic Patient Reported Outcome Measures in Clinical Care: Mixed Methods Study”.


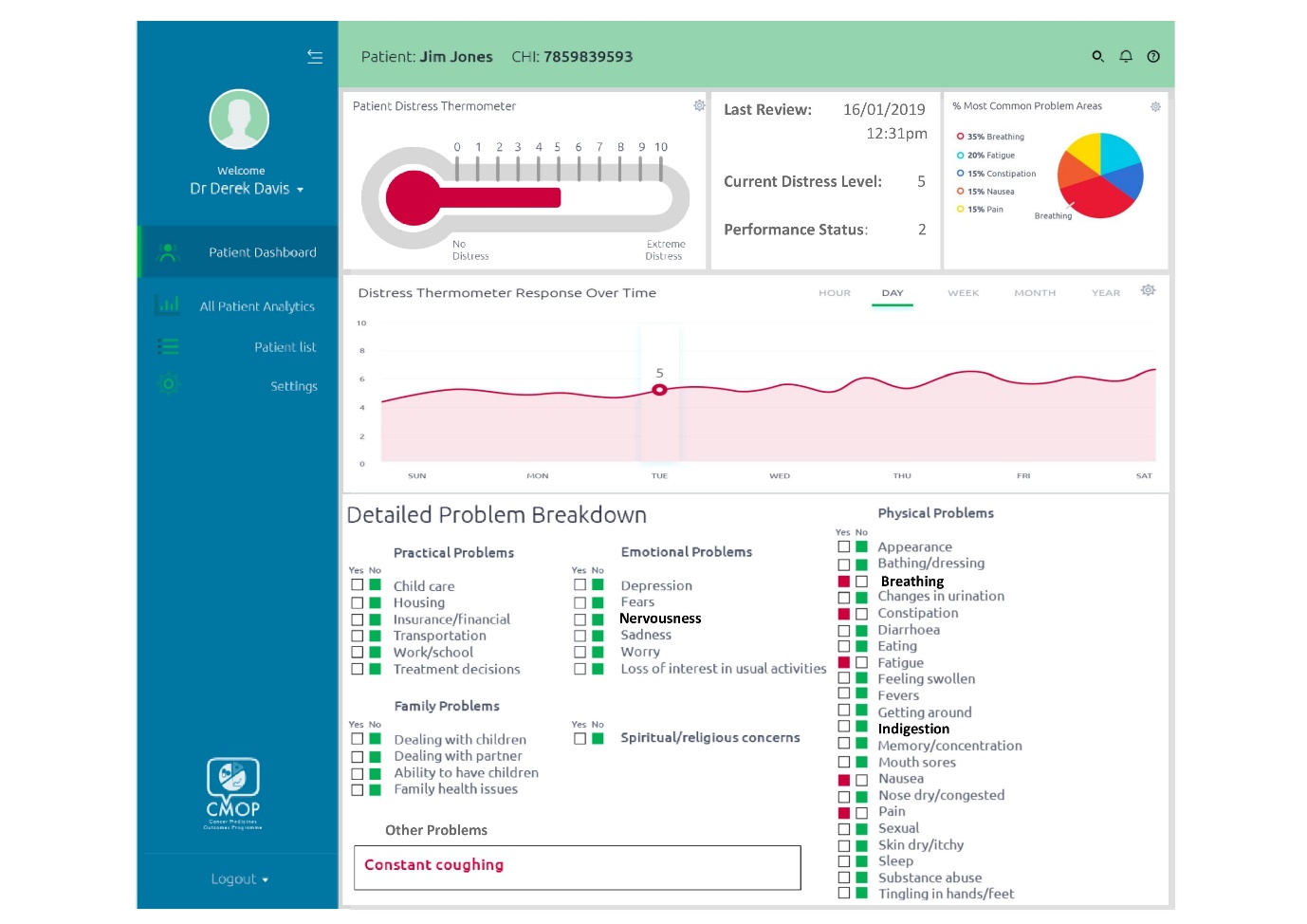

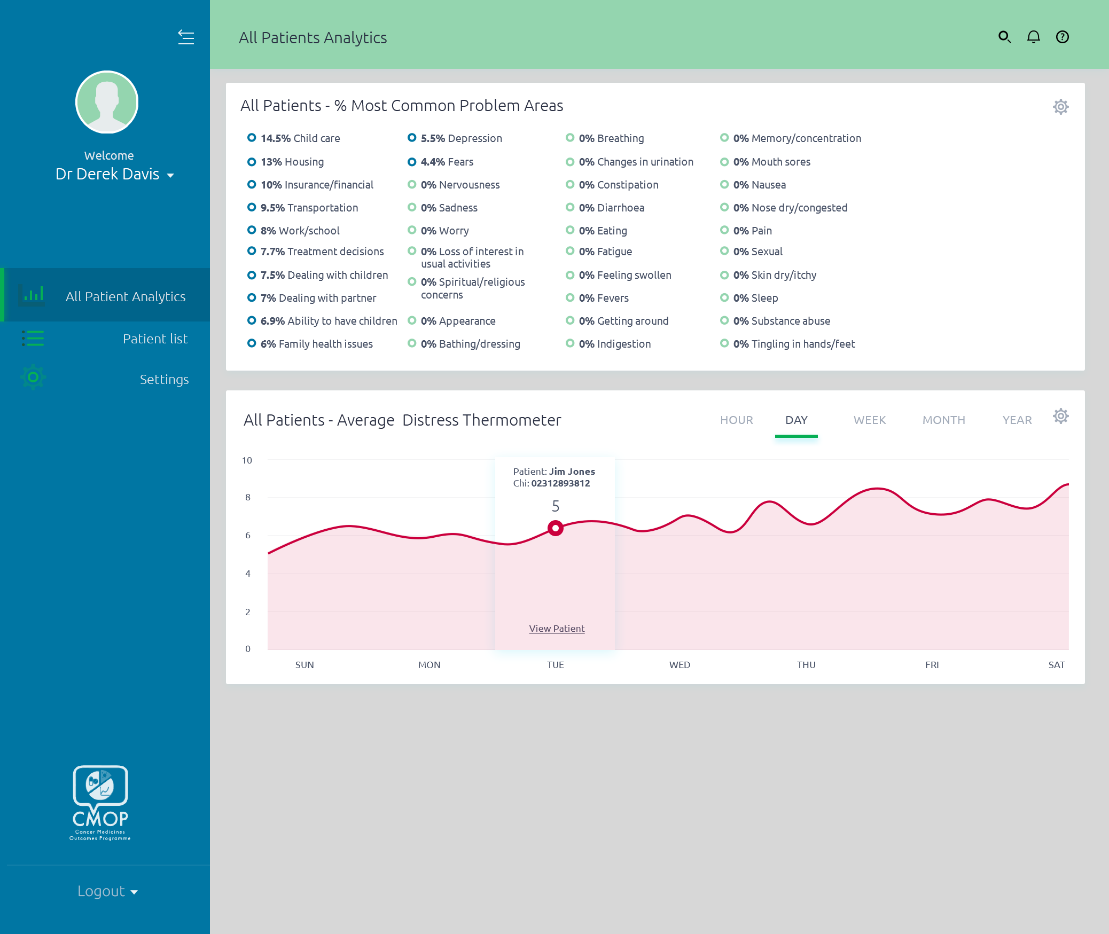


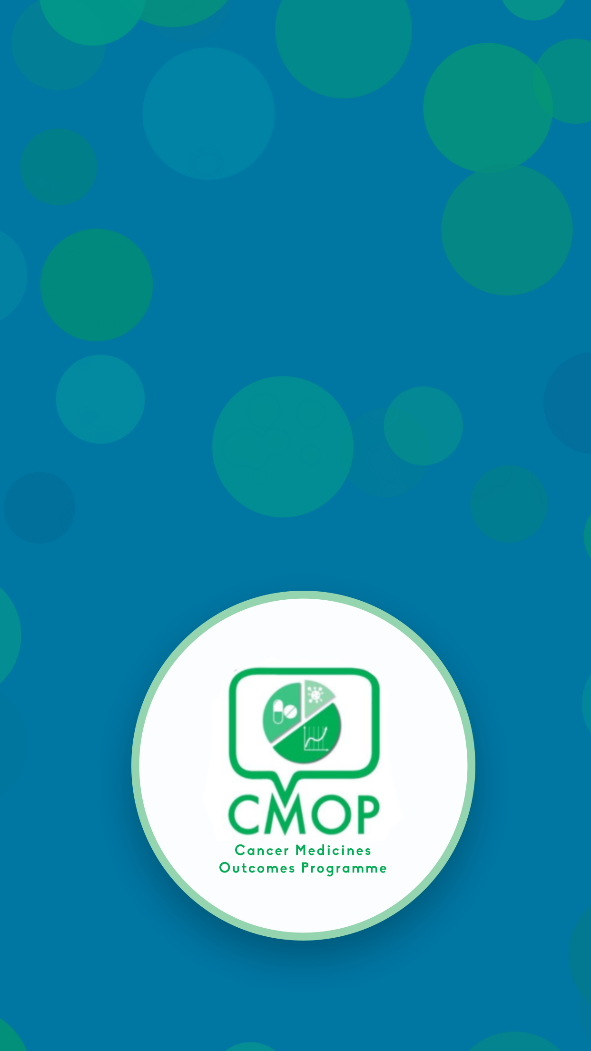

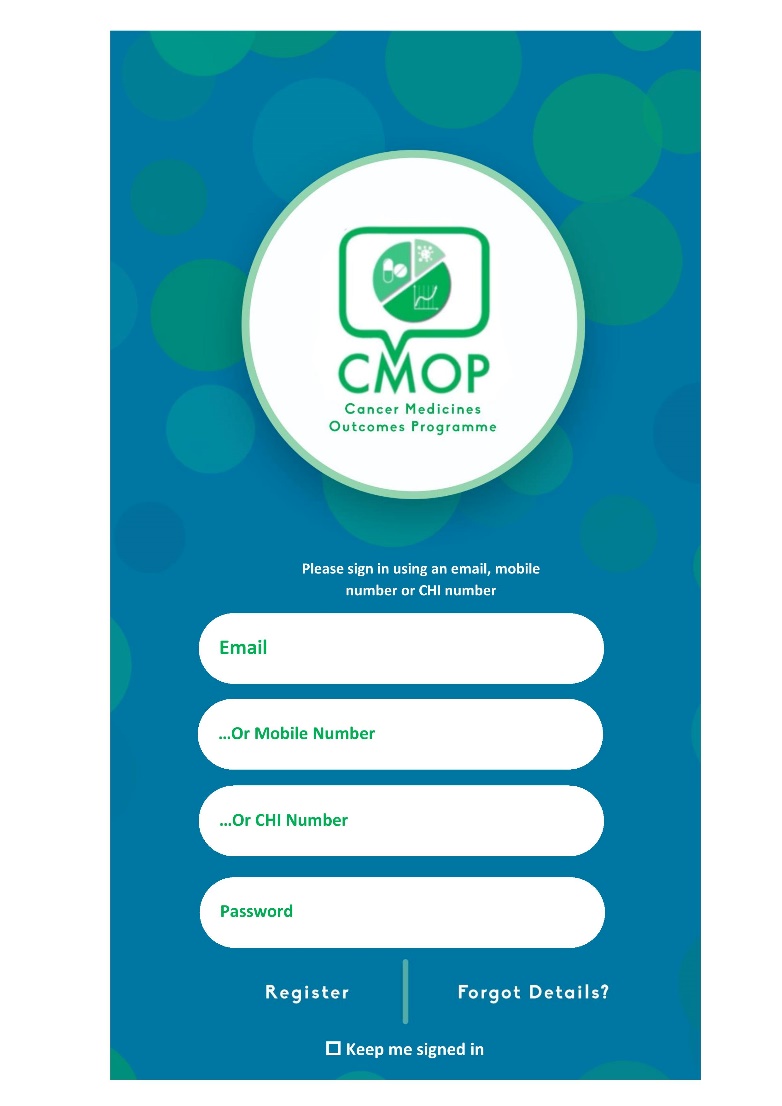

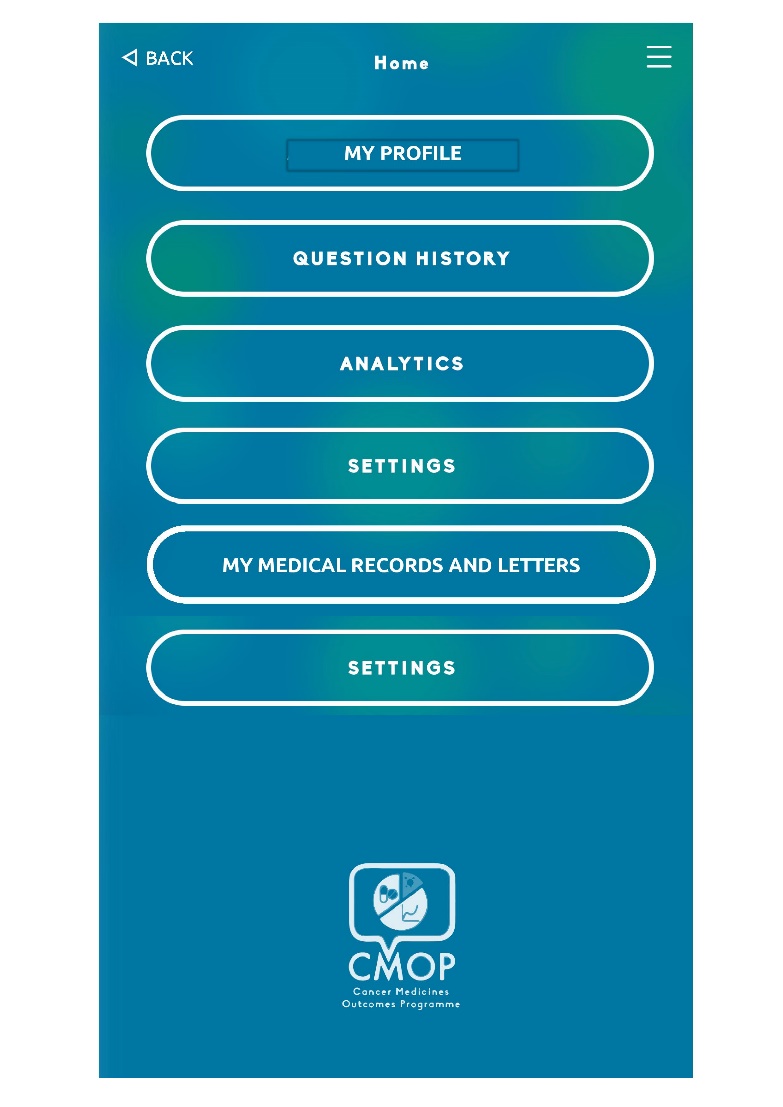

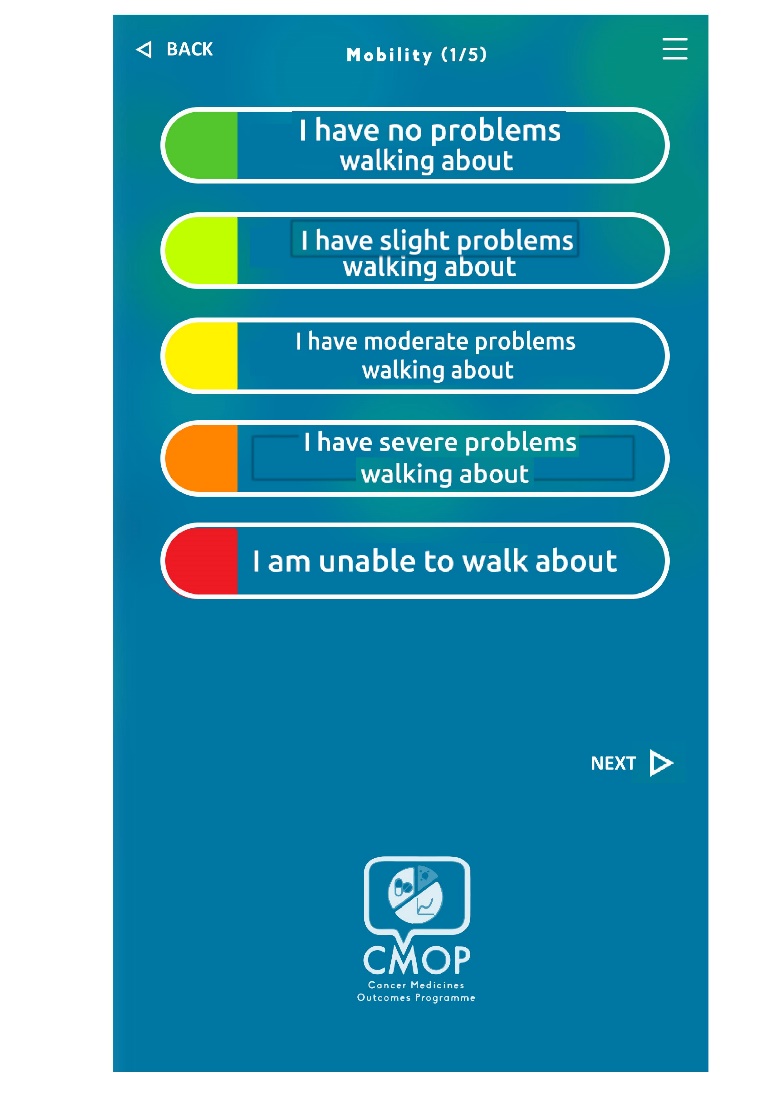

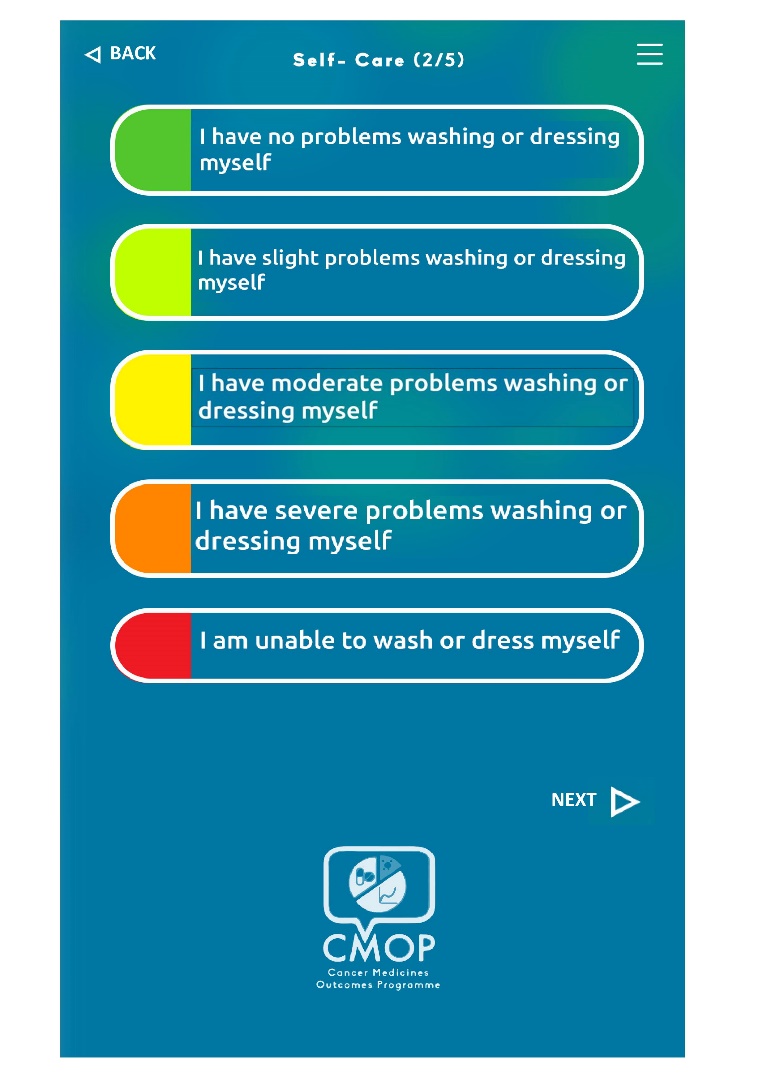

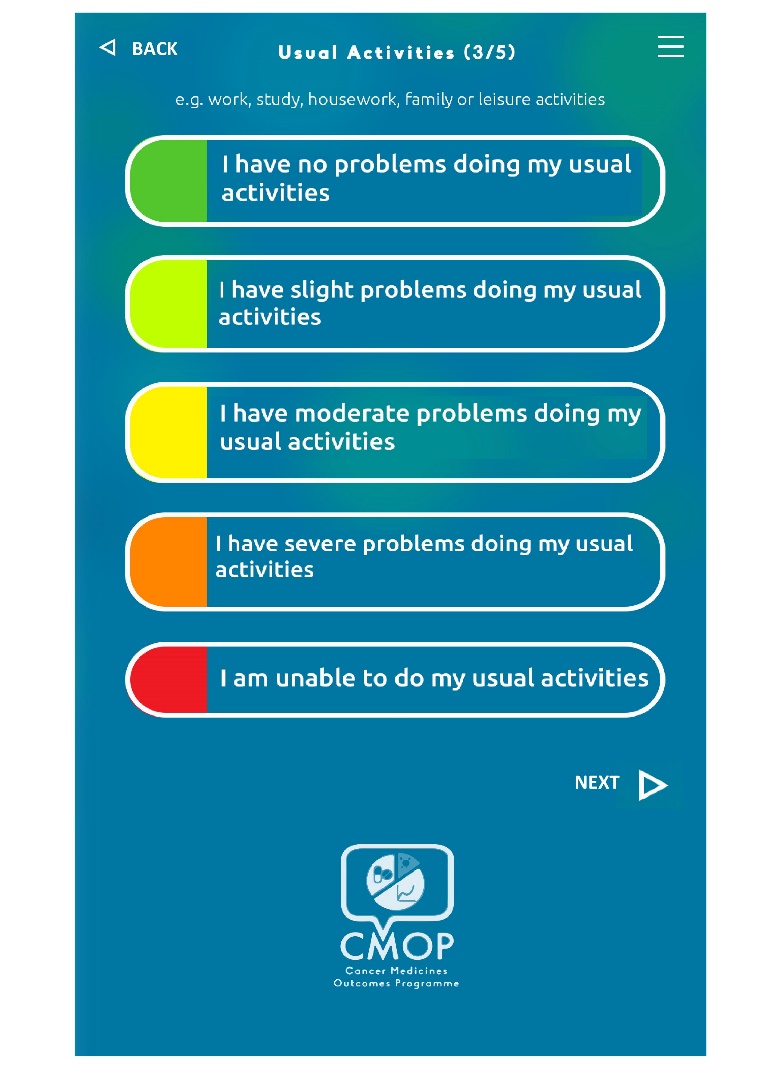

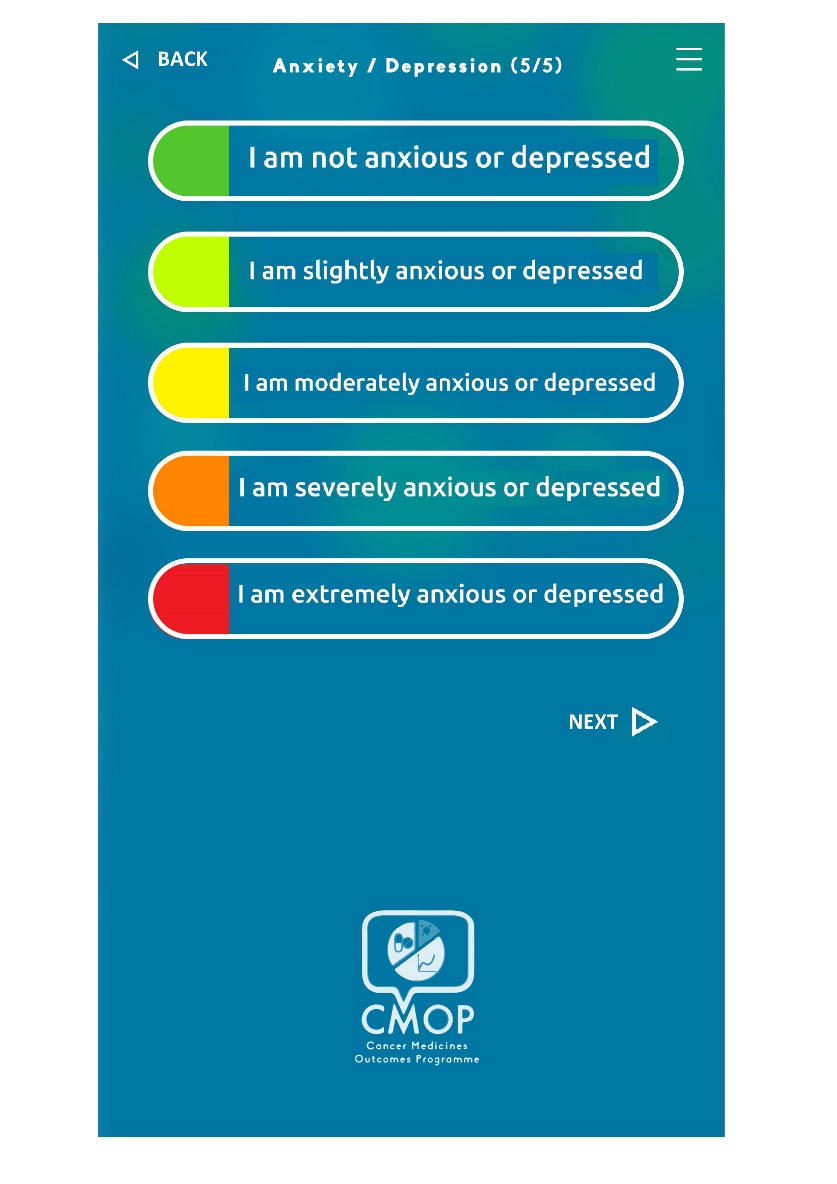

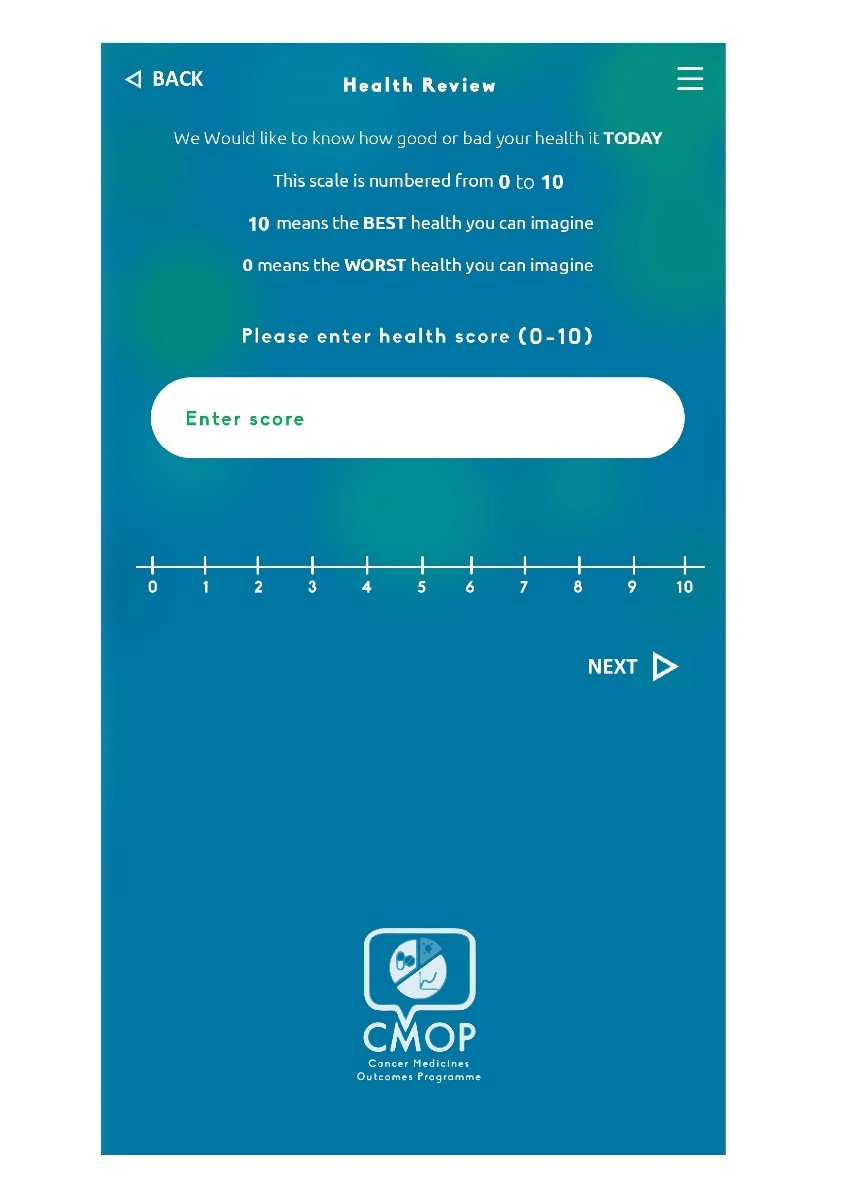

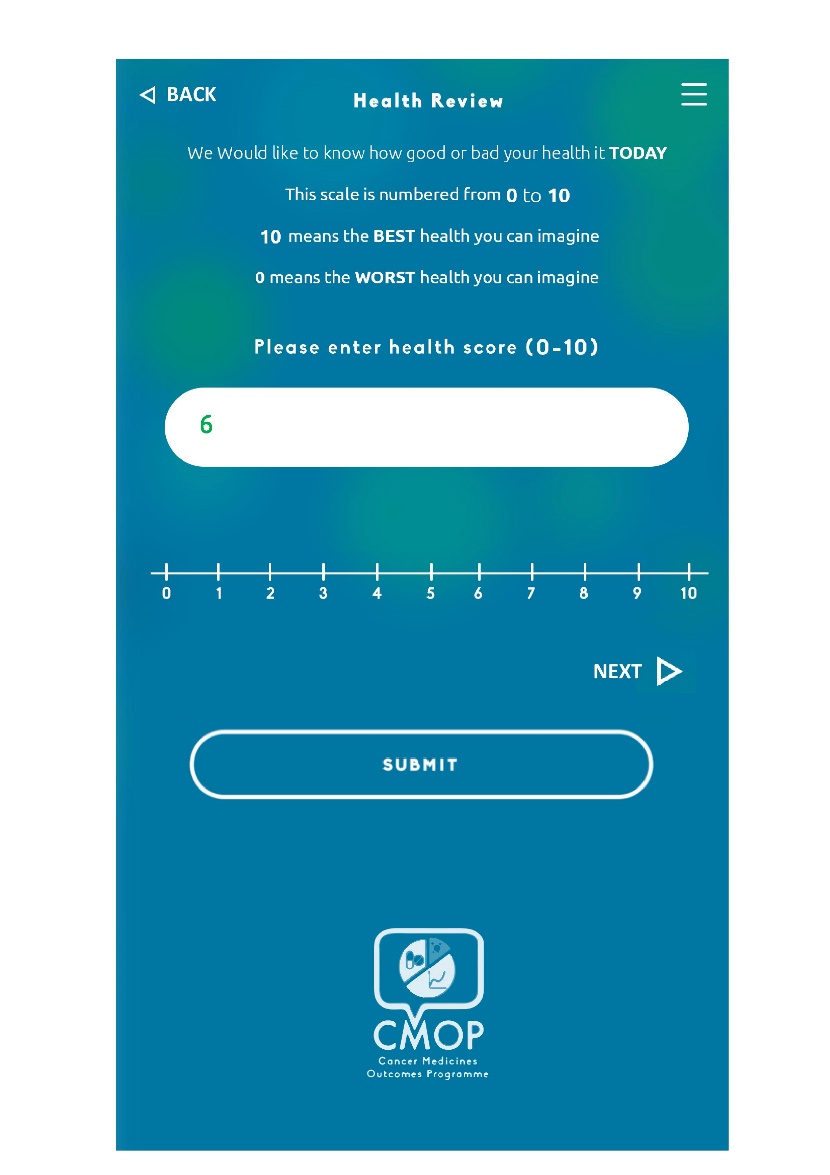

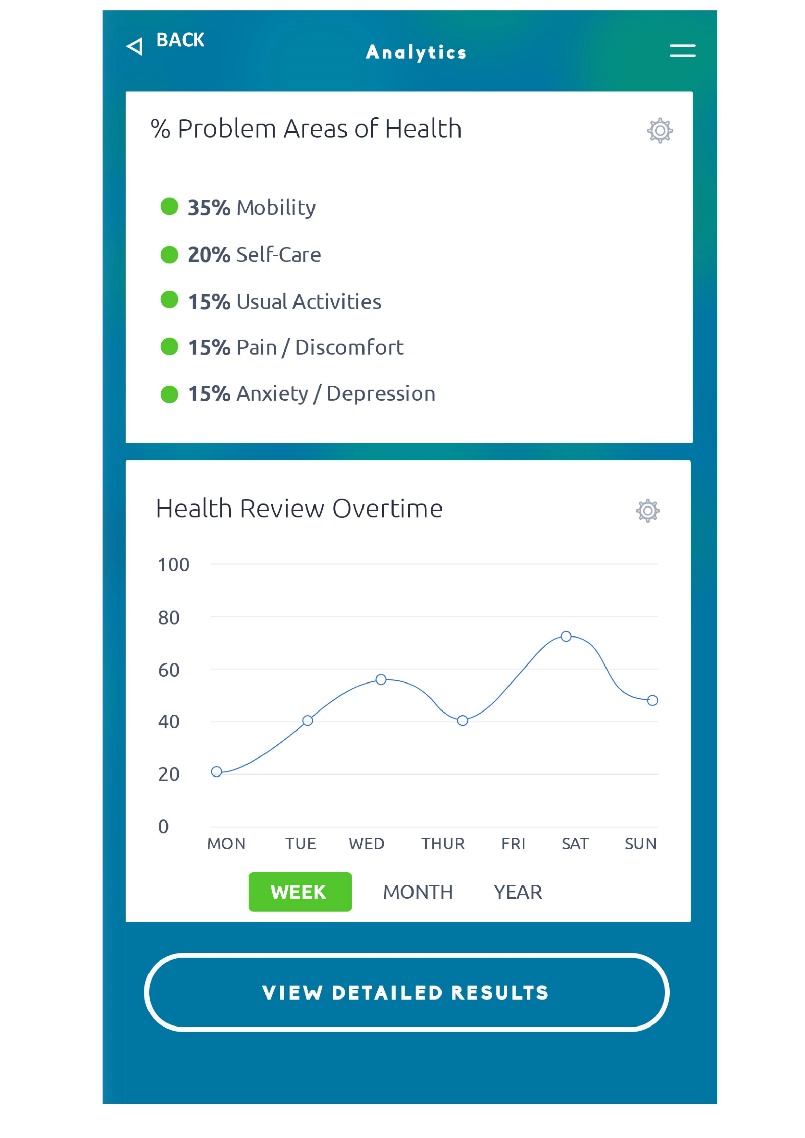

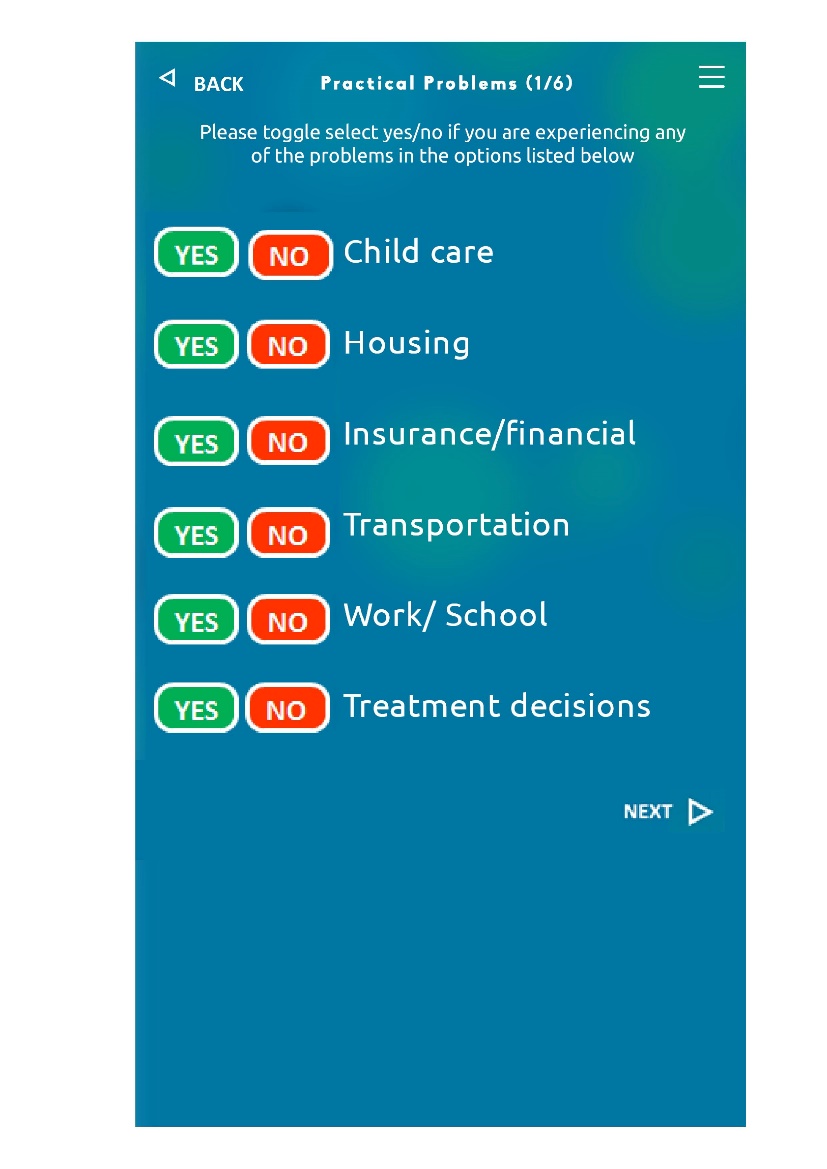

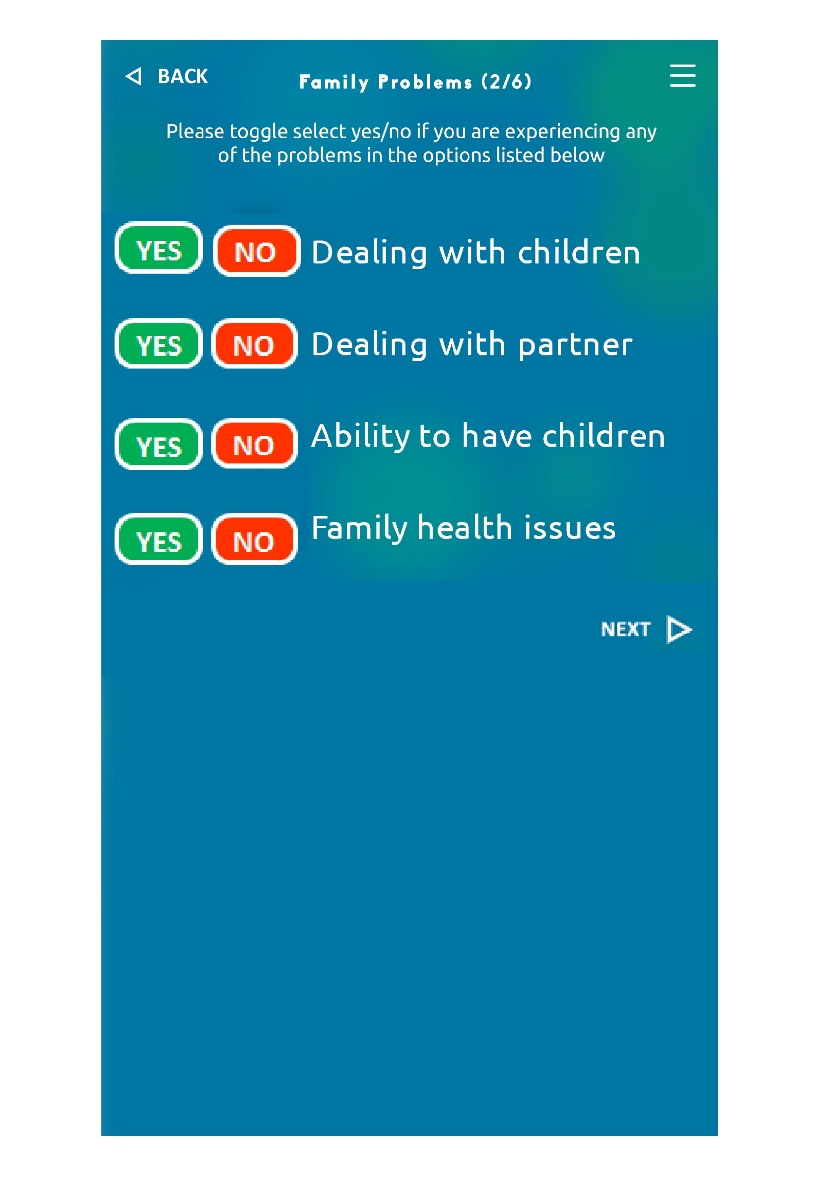

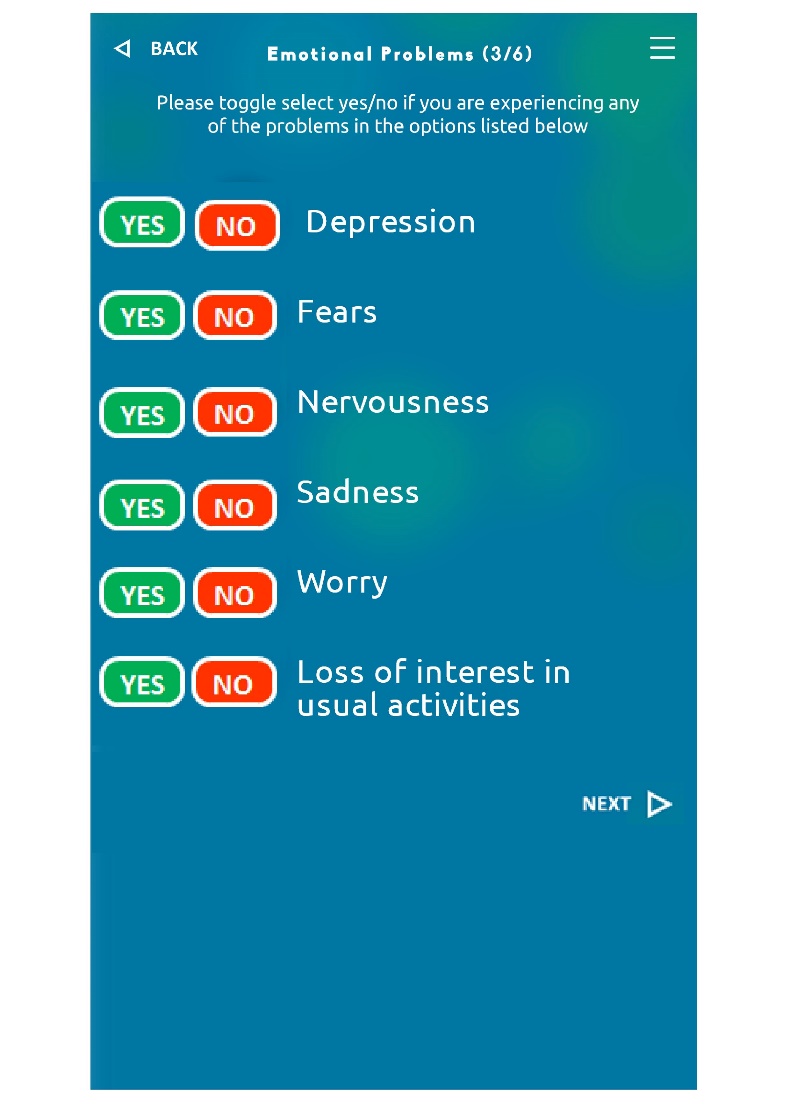

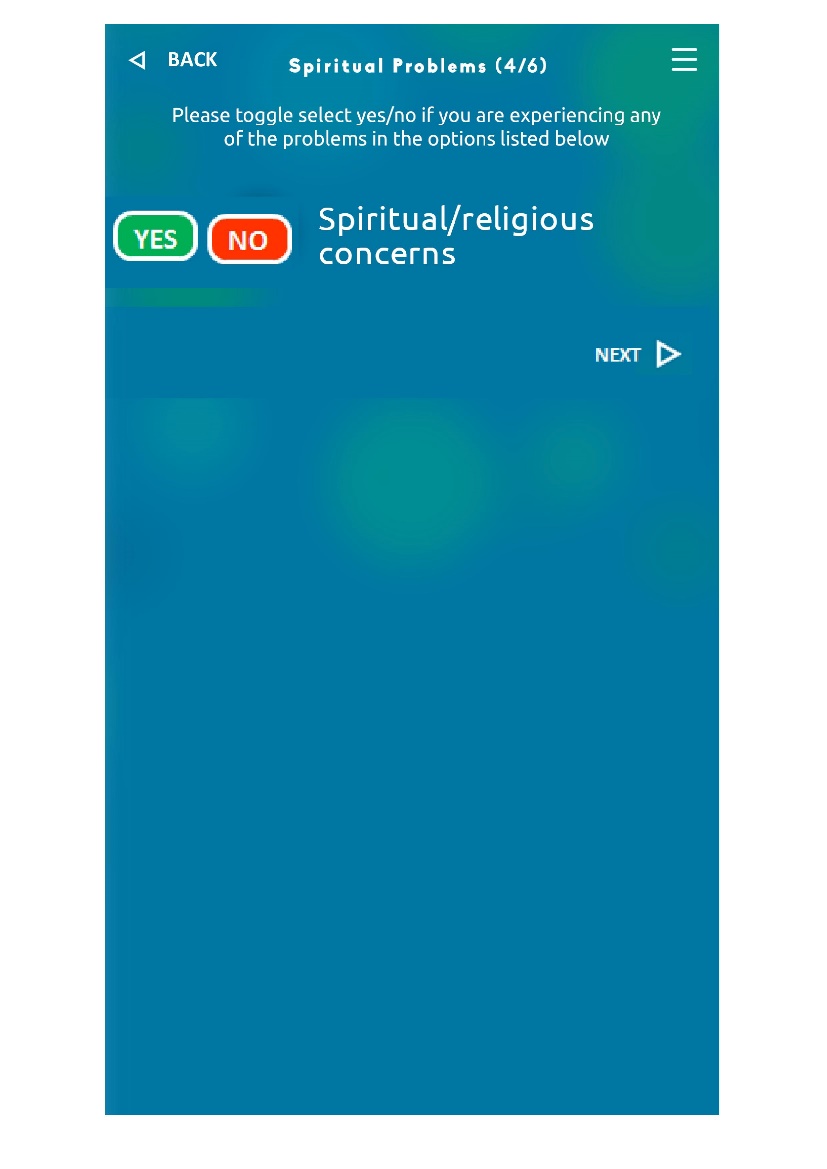

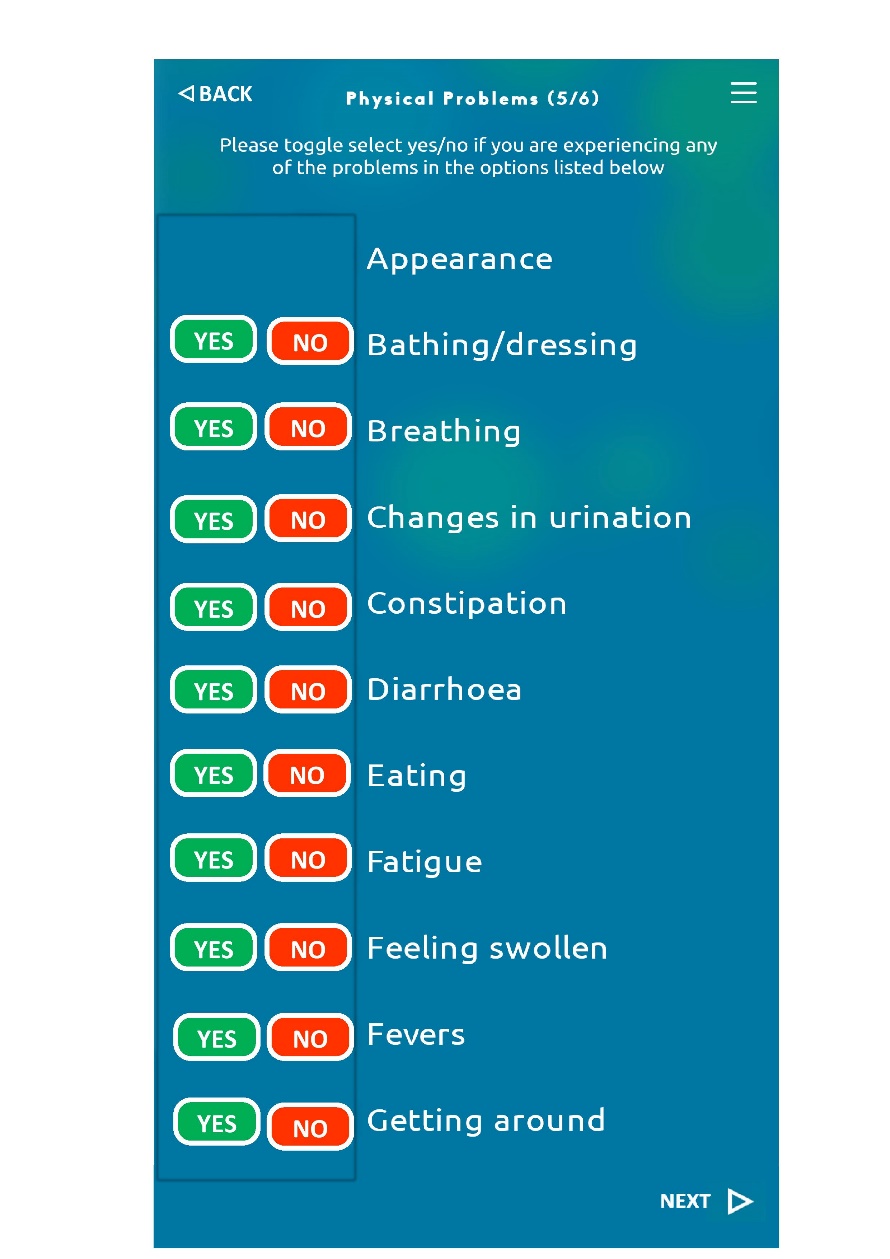

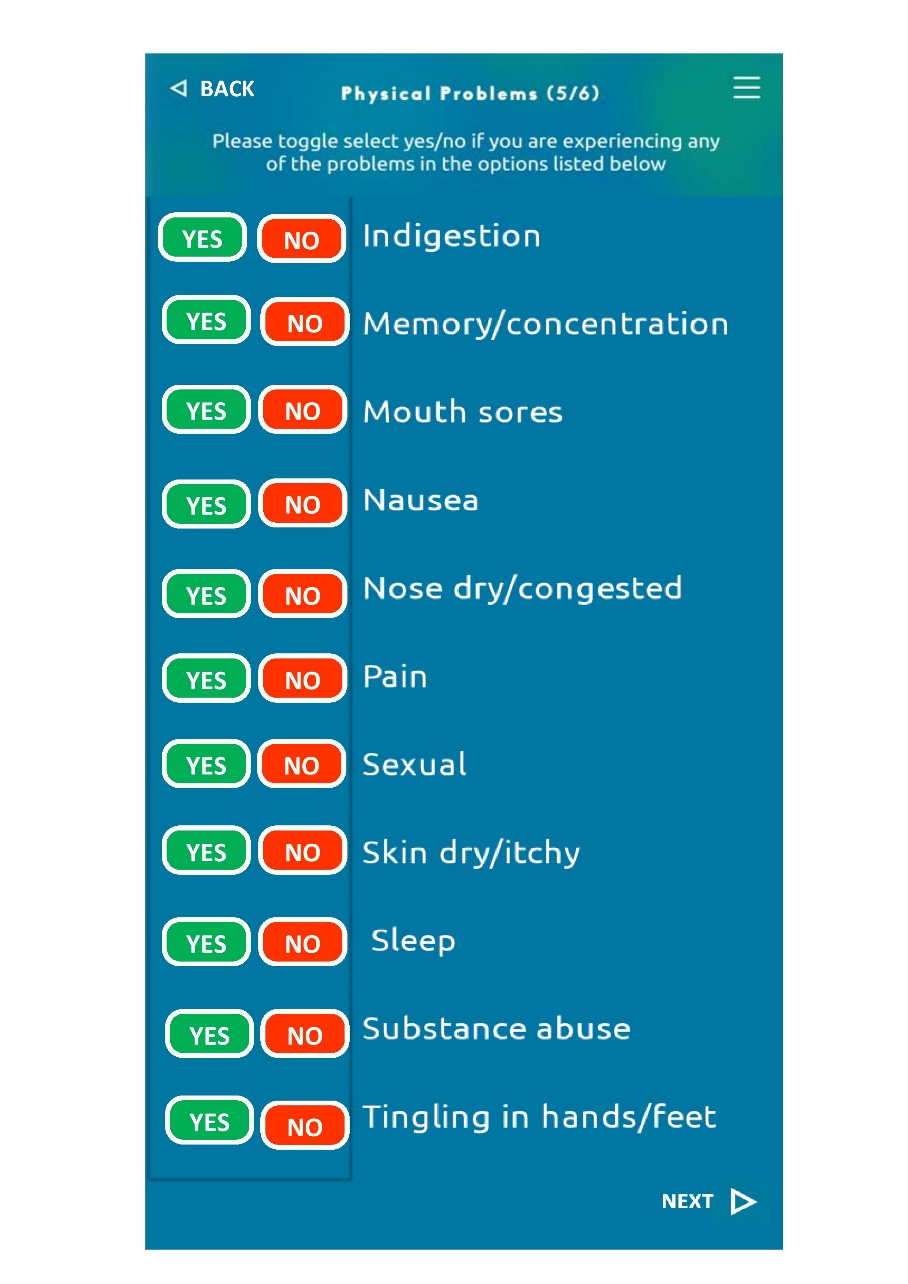

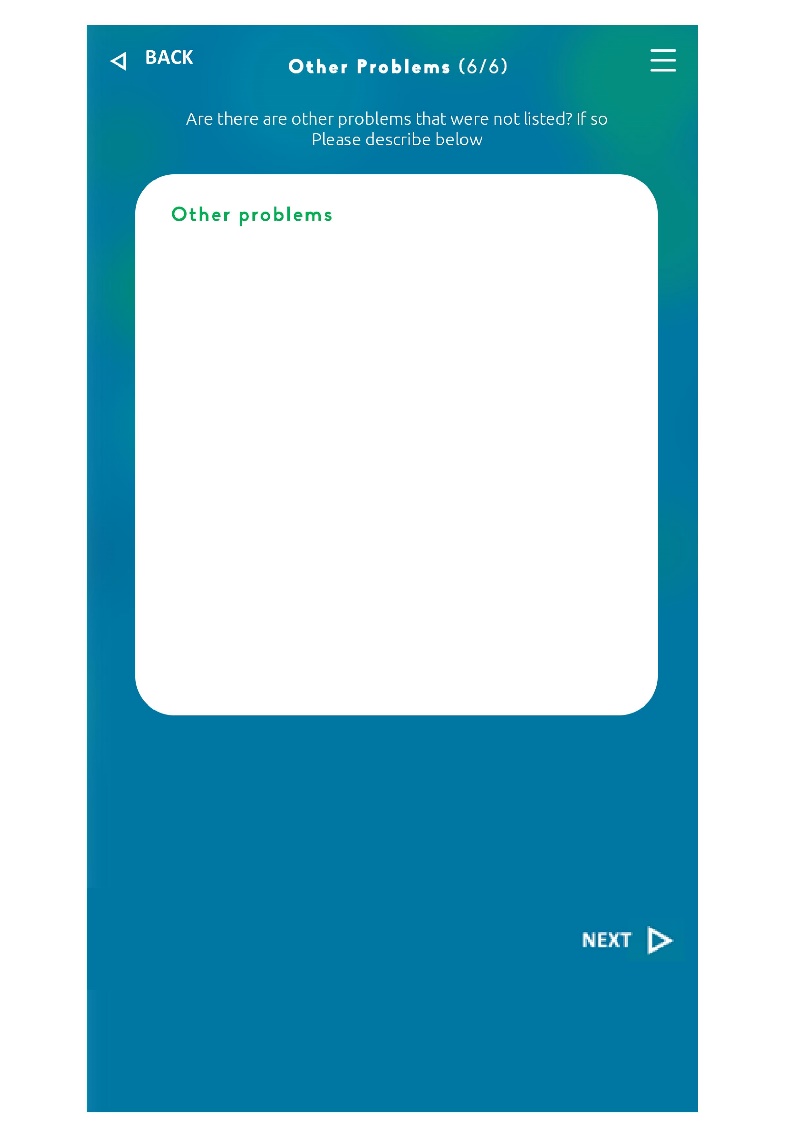

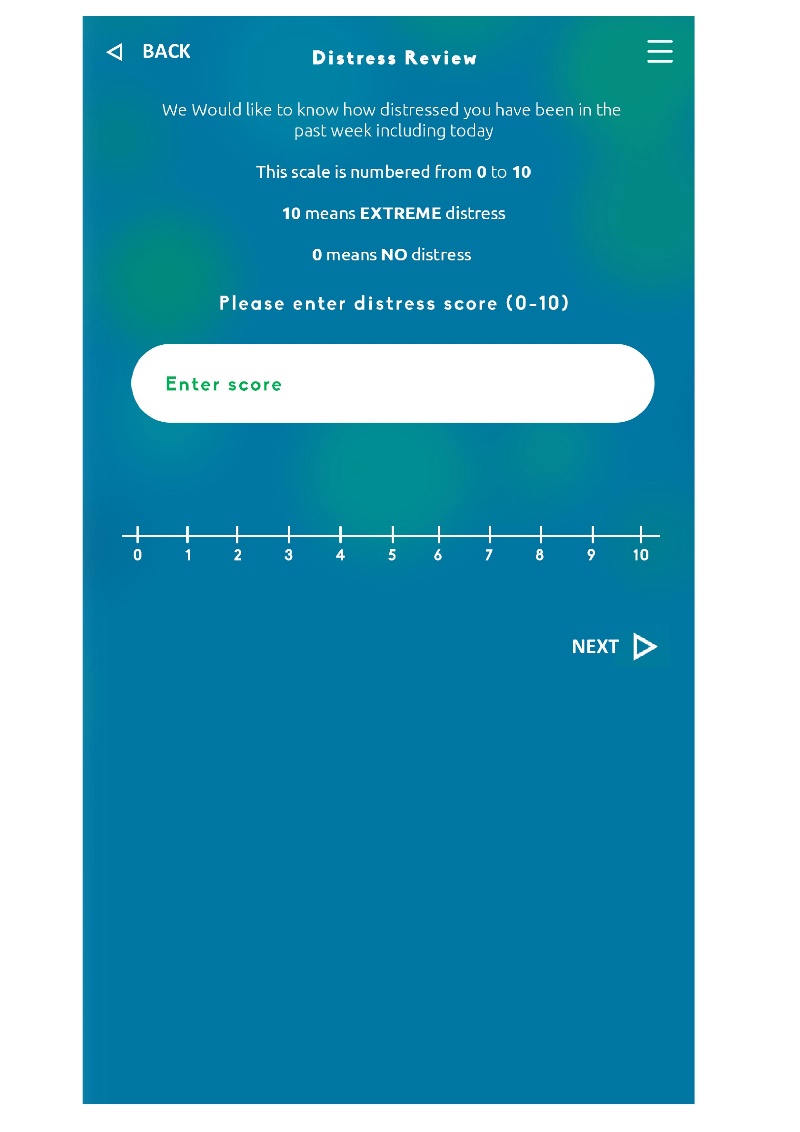

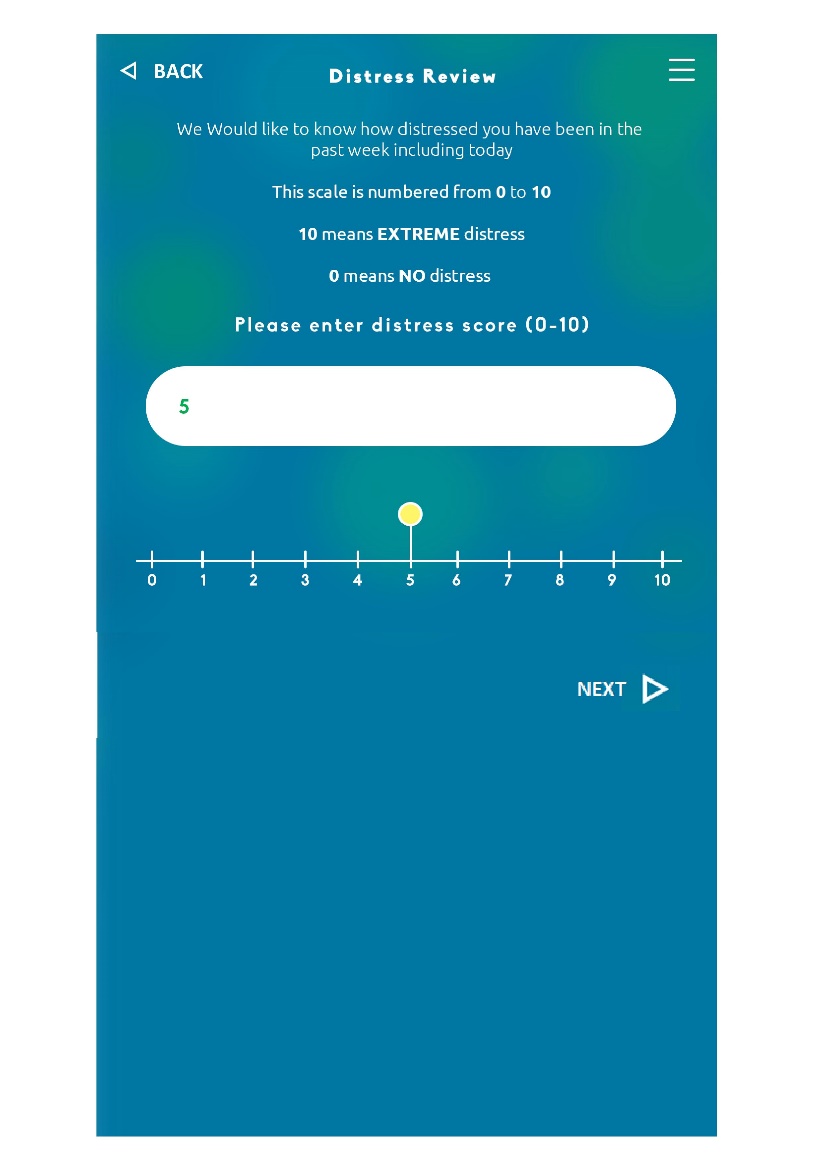

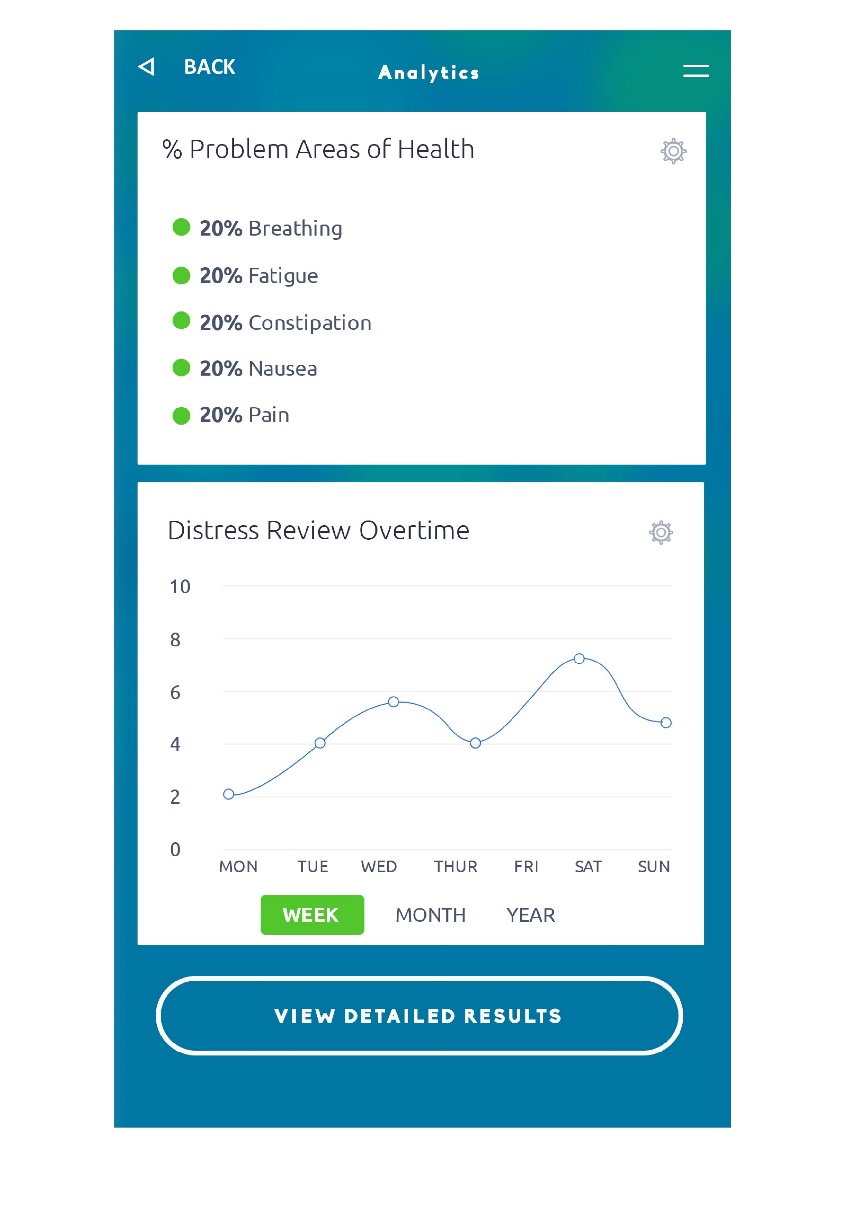

Supplement: Multimedia Appendix 1 [file formative_v7i1e48296_app1.doc]
