# Supplementary material for: Involving Patients and Clinicians in the Design of Wireframes for Cancer Medicines Electronic Patient Reported Outcome Measures in Clinical Care: Mixed Methods Study
Source: JMIR Form Res. 2023 Dec 21;7:e48296. doi: 10.2196/48296 (PMC10767627; doi:10.2196/48296)
Supplement: Multimedia Appendix 2 [file formative_v7i1e48296_app2.docx]

# Multimedia Appendix 2: Description of PROMS tool selection process

This is a Multimedia Appendix 2 for a full manuscript published in JMIR Formative Research. For full copyright and citation information see “Involving Patients and Clinicians in the Design of Wireframes for Cancer Medicines Electronic Patient Reported Outcome Measures in Clinical Care: Mixed Methods Study”.

**Background**

The authors previously conducted a study [38] which aimed to identify what mattered to clinicians and patients when discussing the impact that cancer medicines had on health-related quality of life (HRQoL). In the study, a framework of HRQoL domain/domain elements was developed, informed by the items within published patient reported outcome measures (PROMs) tools. Prostate cancer clinicians and patients ranked their most to least important HRQoL domains elements in relation to how treatment impacts HRQoL. The domain elements important to clinicians and patients (both commonly and exclusively) were identified [38].

Method

The findings from this study were used to Identify which PROMs tool(s) had the greatest number of items that matched the important HRQoL domain elements. The results were presented to the research team which included academics and researchers as well as clinicians working in the area of prostate cancer.

Outcome

Although no single tool could be identified as having items that represented all of the priorities of clinicians and patients, two tools in combination were identified as an acceptable representation of the priorities identified by clinicians and patients:

- National Comprehensive Cancer Network (NCCN) Distress Thermometer & Problems List [39]
- EQ-5D-5L [41].

The items within these tools were then used to populate the content of the app and dashboard wireframes.
