# Supplementary material for: Involving Patients and Clinicians in the Design of Wireframes for Cancer Medicines Electronic Patient Reported Outcome Measures in Clinical Care: Mixed Methods Study
Source: JMIR Form Res. 2023 Dec 21;7:e48296. doi: 10.2196/48296 (PMC10767627; doi:10.2196/48296)
Supplement: Multimedia Appendix 3 [file formative_v7i1e48296_app3.doc]

# Multimedia Appendix 3: CMOP PROMs Dashboard Clinician Interview Schedule

This is a Multimedia Appendix 3 for a full manuscript published in JMIR Formative Research. For full copyright and citation information see “Involving Patients and Clinicians in the Design of Wireframes for Cancer Medicines Electronic Patient Reported Outcome Measures in Clinical Care: Mixed Methods Study”.

*Give introduction*

*Show live version/ Video / projections / paper copies of wireframes*

**INTRODUCTORY QUESTIONS**

1. What kind of PROMs tools, if any, do you currently use or have previously used?

*PROMPT: as part of clinical trial*

*PROMPT: paper questionnaires*

*PROMPT: emails*

*PROMPT: calls*

*PROMPT: own ways of using patient record / notes*

*PROMPT: for select patients based on medication*

*PROMPT: as part of other pilot*

1. Let’s talk about how you discuss quality of life and the impact treatment has with patients

*PROMPT: who brings it up?*

*PROMPT: does it add value?*

*PROMPT: is it easy?*

*PROMPT: does it make a difference right now to treatment choice?*

1. Let’s talk about how you think the dashboard looks.

*PROMPT: colours, fonts, images, appearance*

*PROMPT: quality of images*

*PROMPT: text size / accessibility*

**EASE OF USE**

1. How easy do you think the dashboard would be to work with?

*PROMPT: clarity of instructions*

*PROMPT: clarity of purpose*

*PROMPT: clarity of what all the buttons mean etc.*

*PROMPT: how quickly it would take to navigate and operate it*

1. How much would you like / enjoy working with a dashboard like this?
2. What challenges do you think there would be in you, or other clinicians, using this dashboard?

*PROMPT: access*

*PROMPT: time (in using)*

*PROMPT: time (in setting patients up to use it)*

*PROMPT: age/generation/patient acceptability of the system*

*PROMPT: privacy*

*PROMPT: value to care*

*PROMPT: time, effort*

*PROMPT: confusion / difficult / unclear*

**USEFULNESS**

1. How useful do you think this dashboard would be in the decision-making process?
   1. PROMPT: what benefits would using it maybe have?
2. How do you think patients would respond to an invite to use the app?
   1. PROMPT: hard to recruit
   2. PROMPT: hard to explain purpose
   3. PROMPT: positively, why
3. How would this dashboard make a difference in your care, if at all?

*PROMPT: in making any difference in patient QoL*

*PROMPT: difference in quality of interactions with patients*

*PROMPT: involving patient more in decision making*

*PROMPT: no difference*

*PROMPT: burden*

1. How interested would you be in using this dashboard and app with patients to record how their treatment impacts their QoL?
2. The patient app is designed for patients to complete QoL data a day or 2 before their clinic appointment. How agreeable / acceptable is this for you to see QoL data at every appointment and at this timescale

*PROMPT: too often*

*PROMPT: not often enough*

*PROMPT: issues with patients remembering*

*PROMPT: hassle or burden*

*PROMPT: flexibility*

*PROMPT: complete in-clinic*

**CONCLUDING QUESTIONS**

1. What, if anything, do you really like about this dashboard?
2. What changes / improvements could be made to this dashboard that we haven’t already discussed?
