# Supplementary material for: Involving Patients and Clinicians in the Design of Wireframes for Cancer Medicines Electronic Patient Reported Outcome Measures in Clinical Care: Mixed Methods Study
Source: JMIR Form Res. 2023 Dec 21;7:e48296. doi: 10.2196/48296 (PMC10767627; doi:10.2196/48296)
Supplement: Multimedia Appendix 4 [file formative_v7i1e48296_app4.doc]

# Multimedia Appendix 4: CMOP PROMs App Patient Focus Group Schedule

This is Multimedia Appendix 4 for a full manuscript published in JMIR Formative Research. For full copyright and citation information see “Involving Patients and Clinicians in the Design of Wireframes for Cancer Medicines Electronic Patient Reported Outcome Measures in Clinical Care: Mixed Methods Study”.

*Give introduction*

*Show live version/ video / projections / paper copies of wireframes.*

**INTRODUCTORY QUESTIONS**

1. What kinds of apps, if any, do you use?

*PROMPT: for health*

*PROMPT: mobile phone access*

*PROMPT: day to day vs less often*

*PROMPT: none*

*PROMPT: other ways i.e. websites etc.*

1. Let’s talk about how important, or not, it is to you to communicate with your clinician the impact that your treatment has on your quality of life.

*PROMPT: what value does it have?*

*PROMPT: how easy is it?*

*PROMPT: does it make a difference right now?*

1. Let’s talk about how you think the app looks.

*PROMPT: colours, fonts, images, appearance*

*PROMPT: quality of images*

*PROMPT: text size / accessibility*

**EASE OF USE**

1. How easy do you think the app looks to work?

*PROMPT: clarity of instructions*

*PROMPT: clarity of purpose*

*PROMPT: clarity of what all the buttons mean etc.*

*PROMPT: how quickly it would take to navigate and operate it*

1. How much would you like / enjoy working with an app like this?
2. What challenges do you think there would be in you, or other cancer patients, using this app?

*PROMPT: access to IT*

*PROMPT: age/generation*

*PROMPT: privacy*

*PROMPT: value*

*PROMPT: time, effort*

*PROMPT: confusion / difficult / unclear*

**USEFULNESS**

1. How useful do you think this app would be in communicating with your clinician on how your treatment affects your quality of life?
   1. PROMPT: what benefits would using it maybe have?
2. How would this app make a difference in your care, if at all?

*PROMPT: in making any difference in how my treatment affects my quality of life*

*PROMPT: difference in feeling listened to*

*PROMPT: feeling more involved in decision making*

*PROMPT: no difference*

*PROMPT: care burden*

1. How interested would you be in using this app to record how your treatment impacts your quality of life?
2. This app would be designed for you to complete your quality of life data a day or 2 before your clinic appointment. How agreeable / acceptable is this for you?

*PROMPT: too often*

*PROMPT: not often enough*

*PROMPT: remembering to do it*

*PROMPT: hassle or burden*

*PROMPT: flexibility*

**CONCLUDING QUESTIONS**

1. What, if anything, do you really like about this app?
2. What changes / improvements could be made to this app that we haven’t already discussed?
