# Supplementary material for: Involving Patients and Clinicians in the Design of Wireframes for Cancer Medicines Electronic Patient Reported Outcome Measures in Clinical Care: Mixed Methods Study
Source: JMIR Form Res. 2023 Dec 21;7:e48296. doi: 10.2196/48296 (PMC10767627; doi:10.2196/48296)
Supplement: Multimedia Appendix 5 [file formative_v7i1e48296_app5.doc]

# Multimedia Appendix 5: CMOP PROMs Dashboard Clinician Questionnaire (Stage 2)

This is Multimedia Appendix 5 for a full manuscript published in JMIR Formative Research. For full copyright and citation information see “Involving Patients and Clinicians in the Design of Wireframes for Cancer Medicines Electronic Patient Reported Outcome Measures in Clinical Care: Mixed Methods Study”.

[Participant Information Sheet and Consent Form removed for publication]

Start of Block: ABOUT YOU

Q4 What is your age?

________________________________________________________________

Q5 What is your gender (please tick one)?

- Male
- Female
- Other
- Prefer not to say

Q6 What is your job role (tick one)?

- Consulting Oncologist
- Pharmacist
- Clinical Nurse Specialist
- Other (please specify) ________________________________________________

Q7 At what hospital is the cancer clinic you operate (tick one)?

- The Beatson West of Scotland Cancer Centre
- The New Victoria Infirmary
- Other (please specify) ________________________________________________

Q8 In which cancer area do you work?

________________________________________________________________

Q9 How many years have you been in your current job role?

________________________________________________________________

Q51 Do you currently use any electronic or paper-based Patient Reported Outcome Measures (PROMs) questionnaires or tools with any of your patients (if yes, please elaborate e.g. as part of a clinical trial, name of the questionnaire / tool used etc.)?

No

Yes (please specify) ________________________________________________

End of Block: ABOUT YOU

Start of Block: QUESTIONNAIRE INSTRUCTIONS

Q11 **QUESTIONNAIRE INSTRUCTIONS**
*Please read carefully*   ·

After this page you will be shown mock-ups of a Patient Reported Outcome Measures (PROMs) dashboard, to be embedded within Trakcare **(CMOP PROMs Dashboard)**. This dashboard is designed for you to view the PROMs data that cancer patients record via the CMOP PROMs mobile App on how their treatment affects quality of life.  That quality of life information can be used, along with the patient’s medical test results, to make the best decisions with the patient on their cancer treatment. ​​·

You may have seen previous versions of these mock-ups during a one-to-one interview. Feedback from you and other participants was collated and changes were made. The main changes were:

- Minimise the size of the distress thermometer and the pie chart
- Make the detailed symptoms & side effects section larger
- Indicate when a symptom or side effect is new / there is a change (e.g. bold text) Include performance status

Other suggestions included being able to include questions from specific paper-based tools already used (e.g. chemotherapy booklet) (not reflected in these mock-ups) ·

**This is a pilot version of the CMOP PROMs Dashboard**.

This is just an example. After that, the questionnaire begins. You will be asked to give your opinions on a number of things, like:

- How easy it looks to use
- How clear or professional it looked
- How attractive it was
- If you think you would use it
- If you think it’s a good idea

On the last page, you will be asked to provide any other thoughts on the CMOP PROMs Dashboard, and any benefits or challenges you think using it might pose.

**Please click NEXT to view the mock-ups of the CMOP PROMs Dashboard**

End of Block: QUESTIONNAIRE INSTRUCTIONS

Start of Block: CMOP PROMS DASHBOARD

**[PROMs dashboard Wireframes here (see Supplementary File 1)**

End of Block: CMOP PROMS DASHBOARD

Start of Block: OVERALL EVALUATION

Q12 **I think using the CMOP PROMs dashboard in my job would be: (select one point on all three scales)**

|  | Extremely | Quite | Slightly | Neither | Slightly | Quite | Extremely |  |
| --- | --- | --- | --- | --- | --- | --- | --- | --- |
|  | 1 | 2 | 3 | 4 | 5 | 6 | 7 |  |
| BAD |  |  |  |  |  |  |  | GOOD |
| HARMFUL |  |  |  |  |  |  |  | BENEFICIAL |
| NEGATIVE |  |  |  |  |  |  |  | POSITIVE |

Q13 **Using the scale, how confident are you in the ratings that you have made on this page?**

|  | 1 | 2 | 3 | 4 | 5 | 6 | 7 |  |
| --- | --- | --- | --- | --- | --- | --- | --- | --- |
| Not at all confident |  |  |  |  |  |  |  | Completely confident |

End of Block: OVERALL EVALUATION

Start of Block: PERCIEVED EASE OF USE

Q14 **Learning to operate the CMOP PROMs dashboard would be easy for me.**

|  | Extremely | Quite | Slightly | Neither | Slightly | Quite | Extremely |  |
| --- | --- | --- | --- | --- | --- | --- | --- | --- |
|  | 1 | 2 | 3 | 4 | 5 | 6 | 7 |  |
| Likely |  |  |  |  |  |  |  | Unlikely |

Q15 **I would find it easy to get the CMOP PROMs dashboard to do what I want it to do.**

|  | Extremely | Quite | Slightly | Neither | Slightly | Quite | Extremely |  |
| --- | --- | --- | --- | --- | --- | --- | --- | --- |
|  | 1 | 2 | 3 | 4 | 5 | 6 | 7 |  |
| Likely |  |  |  |  |  |  |  | Unlikely |

Q16 **My interaction with the CMOP PROMs dashboard would be clear and understandable.**

|  | Extremely | Quite | Slightly | Neither | Slightly | Quite | Extremely |  |
| --- | --- | --- | --- | --- | --- | --- | --- | --- |
|  | 1 | 2 | 3 | 4 | 5 | 6 | 7 |  |
| Likely |  |  |  |  |  |  |  | Unlikely |

Q17 **I would find the CMOP PROMs dashboard to be flexible to interact with.**

|  | Extremely | Quite | Slightly | Neither | Slightly | Quite | Extremely |  |
| --- | --- | --- | --- | --- | --- | --- | --- | --- |
|  | 1 | 2 | 3 | 4 | 5 | 6 | 7 |  |
| Likely |  |  |  |  |  |  |  | Unlikely |

Q18 **It would be easy for me to become skillful at using the CMOP PROMs dashboard.**

|  | Extremely | Quite | Slightly | Neither | Slightly | Quite | Extremely |  |
| --- | --- | --- | --- | --- | --- | --- | --- | --- |
|  | 1 | 2 | 3 | 4 | 5 | 6 | 7 |  |
| Likely |  |  |  |  |  |  |  | Unlikely |

Q19 **I would find the CMOP PROMs dashboard easy to use.**

|  | Extremely | Quite | Slightly | Neither | Slightly | Quite | Extremely |  |
| --- | --- | --- | --- | --- | --- | --- | --- | --- |
|  | 1 | 2 | 3 | 4 | 5 | 6 | 7 |  |
| Likely |  |  |  |  |  |  |  | Unlikely |

Q21 **How confident are you in the ratings that you have made on this page?**

|  | 1 | 2 | 3 | 4 | 5 | 6 | 7 |  |
| --- | --- | --- | --- | --- | --- | --- | --- | --- |
| Not at all confident |  |  |  |  |  |  |  | Completely confident |

End of Block: PERCIEVED EASE OF USE

Start of Block: PERCEIVED USEFULNESS

Q22 **Using CMOP PROMs dashboard in my job would enable me to establish the impact treatment has on my patients’ quality of life more quickly.**

|  | Extremely | Quite | Slightly | Neither | Slightly | Quite | Extremely |  |
| --- | --- | --- | --- | --- | --- | --- | --- | --- |
|  | 1 | 2 | 3 | 4 | 5 | 6 | 7 |  |
| Likely |  |  |  |  |  |  |  | Unlikely |

Q23 **Using CMOP PROMs dashboard would improve my ability to make decisions on treatment.**

|  | Extremely | Quite | Slightly | Neither | Slightly | Quite | Extremely |  |
| --- | --- | --- | --- | --- | --- | --- | --- | --- |
|  | 1 | 2 | 3 | 4 | 5 | 6 | 7 |  |
| Likely |  |  |  |  |  |  |  | Unlikely |

Q24 **Using CMOP PROMs dashboard in my job would make my treatment decision making more productive.**

|  | Extremely | Quite | Slightly | Neither | Slightly | Quite | Extremely |  |
| --- | --- | --- | --- | --- | --- | --- | --- | --- |
|  | 1 | 2 | 3 | 4 | 5 | 6 | 7 |  |
| Likely |  |  |  |  |  |  |  | Unlikely |

Q25 **Using CMOP PROMs dashboard would enhance the effectiveness of my treatment decision making.**

|  | Extremely | Quite | Slightly | Neither | Slightly | Quite | Extremely |  |
| --- | --- | --- | --- | --- | --- | --- | --- | --- |
|  | 1 | 2 | 3 | 4 | 5 | 6 | 7 |  |
| Likely |  |  |  |  |  |  |  | Unlikely |

Q26 **Using CMOP PROMs dashboard would make it easier to do my job.**

|  | Extremely | Quite | Slightly | Neither | Slightly | Quite | Extremely |  |
| --- | --- | --- | --- | --- | --- | --- | --- | --- |
|  | 1 | 2 | 3 | 4 | 5 | 6 | 7 |  |
| Likely |  |  |  |  |  |  |  | Unlikely |

Q27 **I would find CMOP PROMs dashboard useful in my job.**

|  | Extremely | Quite | Slightly | Neither | Slightly | Quite | Extremely |  |
| --- | --- | --- | --- | --- | --- | --- | --- | --- |
|  | 1 | 2 | 3 | 4 | 5 | 6 | 7 |  |
| Likely |  |  |  |  |  |  |  | Unlikely |

Q28 **How confident are you in the ratings that you have made on this page?**

|  | 1 | 2 | 3 | 4 | 5 | 6 | 7 |  |
| --- | --- | --- | --- | --- | --- | --- | --- | --- |
| Not at all confident |  |  |  |  |  |  |  | Completely confident |

End of Block: PERCEIVED USEFULNESS

Start of Block: ANTICIPATED USE OF THE CMOP PROMs DASHBOARD

Q29 **For questions on this page, assume the CMOP PROMs dashboard would be available for you to use on your current job when engaging with patients in the clinic and making decision on their treatment.**

Q30 **Assuming CMOP PROMs dashboard would be available on my job, I predict that I will use it on a regular basis in the future.**

|  | Extremely | Quite | Slightly | Neither | Slightly | Quite | Extremely |  |
| --- | --- | --- | --- | --- | --- | --- | --- | --- |
|  | 1 | 2 | 3 | 4 | 5 | 6 | 7 |  |
| Likely |  |  |  |  |  |  |  | Unlikely |
| Probable |  |  |  |  |  |  |  | Improbable |

Q32 **In my job, I am most likely to use CMOP PROMs dashboard (pick one):**

- With none of my patients
- With a small proportion of my patients
- With about half of my patients
- With most of my patients
- With all of my patients

Q33 **I would recommend using the CMOP PROMs dashboard and patient app package to other clinicians to view patient quality of life data, so that information can be used in clinical decision making better.**

|  | Extremely | Quite | Slightly | Neither | Slightly | Quite | Extremely |  |
| --- | --- | --- | --- | --- | --- | --- | --- | --- |
|  | 1 | 2 | 3 | 4 | 5 | 6 | 7 |  |
| Likely |  |  |  |  |  |  |  | Unlikely |

Q34 **What are the chances in 100 that you will use the CMOP PROMs dashboard in clinic with patients to enable you to make decisions on their treatment?**

|  | 0 | 10 | 20 | 30 | 40 | 50 | 60 | 70 | 80 | 90 | 100 |
| --- | --- | --- | --- | --- | --- | --- | --- | --- | --- | --- | --- |

| Use the slider to indicate () | 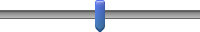 |
| --- | --- |

Q35 **How confident are you in the ratings that you have made on this page?**

|  | 1 | 2 | 3 | 4 | 5 | 6 | 7 |  |
| --- | --- | --- | --- | --- | --- | --- | --- | --- |
| Not at all confident |  |  |  |  |  |  |  | Completely confident |

End of Block: ANTICIPATED USE OF THE CMOP PROMs DASHBOARD

Start of Block: PERCEIVED CHARACTERISTICS OF OUTPUT

Q36 **Assuming I were to use CMOP PROMs dashboard, the quality of my decisions on treatment choice and the care provided would be high.**

|  | Extremely | Quite | Slightly | Neither | Slightly | Quite | Extremely |  |
| --- | --- | --- | --- | --- | --- | --- | --- | --- |
|  | 1 | 2 | 3 | 4 | 5 | 6 | 7 |  |
| Likely |  |  |  |  |  |  |  | Unlikely |

Q37 **Using CMOP PROMs dashboard, the effectiveness of my decision making and the care provided would be:**

|  | Extremely | Quite | Slightly | Neither | Slightly | Quite | Extremely |  |
| --- | --- | --- | --- | --- | --- | --- | --- | --- |
|  | 1 | 2 | 3 | 4 | 5 | 6 | 7 |  |
| LOW |  |  |  |  |  |  |  | HIGH |

Q38 **How confident are you in the ratings that you have made on this page?**

|  | 1 | 2 | 3 | 4 | 5 | 6 | 7 |  |
| --- | --- | --- | --- | --- | --- | --- | --- | --- |
| Not at all confident |  |  |  |  |  |  |  | Completely confident |

End of Block: PERCEIVED CHARACTERISTICS OF OUTPUT

Start of Block: ANTICIPATED ENJOYMENT OF USING THE CMOP PROMs DASHBOARD

Q39 **I would find using the CMOP PROMs dashboard to be enjoyable.**

|  | Extremely | Quite | Slightly | Neither | Slightly | Quite | Extremely |  |
| --- | --- | --- | --- | --- | --- | --- | --- | --- |
|  | 1 | 2 | 3 | 4 | 5 | 6 | 7 |  |
| Likely |  |  |  |  |  |  |  | Unlikely |

Q40 **Using the CMOP PROMs dashboard would be:**

|  | Extremely | Quite | Slightly | Neither | Slightly | Quite | Extremely |  |
| --- | --- | --- | --- | --- | --- | --- | --- | --- |
|  | 1 | 2 | 3 | 4 | 5 | 6 | 7 |  |
| Pleasant |  |  |  |  |  |  |  | Unpleasant |

Q41 **How confident are you in the ratings that you have made on this page?**

|  | 1 | 2 | 3 | 4 | 5 | 6 | 7 |  |
| --- | --- | --- | --- | --- | --- | --- | --- | --- |
| Not at all confident |  |  |  |  |  |  |  | Completely confident |

End of Block: ANTICIPATED ENJOYMENT OF USING THE CMOP PROMs DASHBOARD

Start of Block: PATIENT REPORTED OUTCOME MEASURE DATA

Q42 **In my job, Patient Reported Outcome Measures (PROMs) are (select one point for each statement):**

|  | Extremely | Quite | Slightly | Neither | Slightly | Quite | Extremely |  |
| --- | --- | --- | --- | --- | --- | --- | --- | --- |
|  | 1 | 2 | 3 | 4 | 5 | 6 | 7 |  |
| Unimportant |  |  |  |  |  |  |  | Important |
| Relevant |  |  |  |  |  |  |  | Irrelevant |

Q43 **I would rate the expected difficulty of using the CMOP PROMs dashboard as (use the slider):**

|  | Impossible | Moderate Effort | Effortless |
| --- | --- | --- | --- |

|  |  | 0 | 1 | 2 | 3 | 4 | 5 | 6 | 7 |
| --- | --- | --- | --- | --- | --- | --- | --- | --- | --- |

| Please use the slider () | 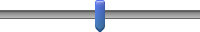 |
| --- | --- |

Q44 **I would rate the quality of the CMOP PROMs dashboard as (use the slider):**

|  | Unacceptable (or non-existent) | Moderate Quality | Professional Quality |
| --- | --- | --- | --- |

|  |  | 0 | 1 | 2 | 3 | 4 | 5 | 6 | 7 |
| --- | --- | --- | --- | --- | --- | --- | --- | --- | --- |

| Please use the slider () | 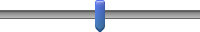 |
| --- | --- |

End of Block: PATIENT REPORTED OUTCOME MEASURE DATA

Start of Block: OTHER BENEFITS OR CHALLENGES

Q45 We would like to know if you can think of any other benefits or challenges to using the CMOP PROMs Dashboard. Please type them in the appropriate boxes below.

Q46 **Benefits of Using the CMOP PROMs Dashboard**

________________________________________________________________

Q47 **Challenges of Using the CMOP PROMs Dashboard**

________________________________________________________________

End of Block: OTHER BENEFITS OR CHALLENGES

Start of Block: THANK YOU

**[Thank you statement and researcher contact details removed for publication]**
