# Supplementary material for: Involving Patients and Clinicians in the Design of Wireframes for Cancer Medicines Electronic Patient Reported Outcome Measures in Clinical Care: Mixed Methods Study
Source: JMIR Form Res. 2023 Dec 21;7:e48296. doi: 10.2196/48296 (PMC10767627; doi:10.2196/48296)
Supplement: Multimedia Appendix 6 [file formative_v7i1e48296_app6.doc]

# Multimedia Appendix 6: CMOP PROMs App Patient Questionnaire (Stage 2)

This is Multimedia Appendix 6 for a full manuscript published in JMIR Formative Research. For full copyright and citation information see “Involving Patients and Clinicians in the Design of Wireframes for Cancer Medicines Electronic Patient Reported Outcome Measures in Clinical Care: Mixed Methods Study”.

**[Participant Information Sheet and Consent removed for publication]**

Start of Block: ABOUT YOU

What is your age?

________________________________________________________________

At what hospital is the cancer clinic you attend (*tick one*)?

- The Beatson West of Scotland Cancer Centre
- The New Victoria Infirmary

Are you a cancer patient, or a family member / carer of someone with cancer (*tick one*)?

- I am a cancer patient
- I am a family member / carer of someone with cancer
- I do not have / do not care for someone with cancer (if so, please stop completing this questionnaire)

What cancer diagnosis have you (or the person you care for) received (*tick one*)?

- Prostate cancer
- Melanoma
- Cervical cancer
- Vulval cancer
- Endometrial cancer
- Other (please specify) ________________________________________________

What is your gender (please tick one)?

- Male
- Female
- Other
- Prefer not to say

When did you / the person you care for receive a cancer diagnosis (*tick one*)?

- Less than 6 months ago
- Between 6 months and 1 year ago
- Between 1 and 5 years ago
- More than 5 years ago
- I don't know / not sure

What treatment are you (or the person you care for) **currently** **receiving for cancer** (*tick all that apply*)?

- Chemotherapy or immunotherapy by injection / 'drip' at hospital
- Tablets / capsules prescribed by hospital
- Regular hormone injections at my GP surgery monthly, 3-monthly or 6-monthly
- Hormone tablets
- Steroids
- Radiotherapy (delivered by a machine)
- Other (please write) ____________________________________________
- I'm not sure
- None of these

What treatments have you (or the person you care for) received **in the past for cancer** (*tick all that apply*):

- Chemotherapy or immunotherapy by injection / 'drip' at hospital
- Tablets / capsules prescribed by hospital
- Regular hormone injections at my GP surgery monthly, 3-monthly or 6-monthly
- Hormone tablets
- Steroids
- Radiotherapy (delivered by a machine)
- Other (please write) ________________________________________________
- I'm not sure
- None of these

How frequently do you (or the person you care for) attend your clinic (*tick one*)?

- Every 3 to 4 weeks
- Every 3 months
- Every 6 months
- Once a year

End of Block: ABOUT YOU

Start of Block: QUESTIONNAIRE INSTRUCTIONS

**QUESTIONNAIRE INSTRUCTIONS**

*Please read carefully*

After this page you will be shown a pilot or test version of a patient mobile app, the **CMOP PROMs App**. This app is designed for cancer patients and/or family members/friends who care for someone with cancers to record how their treatment affects the patient’s quality of life.  The information would then go directly into the patient record for cancer clinicians to see when patients attend their clinic appointment. That quality of life information would then be used, along with the patient’s medical test results, to make the best decisions with the patient on their cancer treatment.   ·

**Please look at all of the mock-ups of the CMOP PROMs App pages- you will need to scroll down the page. Once you have seen them all, click the next button.**   ·

After that, the questionnaire begins. You will be asked to give your opinions on a number of things, like:

- How easy it looks to use
- How clear or professional it looked
- How attractive it was
- If you think you would use it
- If you think it’s a good idea

On the last page, you will be asked to provide any other thoughts on the CMOP PROMs App, and any benefits or challenges you think using it might pose.

**Please click NEXT to visit the pilot version of the CMOP PROMs App**

End of Block: QUESTIONNAIRE INSTRUCTIONS

Start of Block: CMOP PROMs app

____________________________________________________________________________

[App wireframes displayed, see Multimedia Appendix 1)

End of Block: CMOP PROMS APP

Start of Block: OVERALL EVALUATION

**I think using the CMOP PROMs app would be: (select one option per scale)**

|  | Extremely | Quite | Slightly | Neither | Slightly | Quite | Extremely |  |
| --- | --- | --- | --- | --- | --- | --- | --- | --- |
|  | 1 | 2 | 3 | 4 | 5 | 6 | 7 |  |
| BAD |  |  |  |  |  |  |  | GOOD |
| HARMFUL |  |  |  |  |  |  |  | BENEFICIAL |
| NEGATIVE |  |  |  |  |  |  |  | POSITIVE |

**Using the scale, how confident are you in the ratings that you have made on this page?**

|  | 1 | 2 | 3 | 4 | 5 | 6 | 7 |  |
| --- | --- | --- | --- | --- | --- | --- | --- | --- |
| Not at all confident |  |  |  |  |  |  |  | Completely confident |

End of Block: OVERALL EVALUATION

Start of Block: PERCIEVED EASE OF USE

**Learning to operate the CMOP PROMs app would be easy for me.**

|  | Extremely | Quite | Slightly | Neither | Slightly | Quite | Extremely |  |
| --- | --- | --- | --- | --- | --- | --- | --- | --- |
|  | 1 | 2 | 3 | 4 | 5 | 6 | 7 |  |
| Likely |  |  |  |  |  |  |  | Unlikely |

**I would find it easy to get the CMOP PROMs app to do what I want it to do.**

|  | Extremely | Quite | Slightly | Neither | Slightly | Quite | Extremely |  |
| --- | --- | --- | --- | --- | --- | --- | --- | --- |
|  | 1 | 2 | 3 | 4 | 5 | 6 | 7 |  |
| Likely |  |  |  |  |  |  |  | Unlikely |

**My interaction with the CMOP PROMs app would be clear and understandable.**

|  | Extremely | Quite | Slightly | Neither | Slightly | Quite | Extremely |  |
| --- | --- | --- | --- | --- | --- | --- | --- | --- |
|  | 1 | 2 | 3 | 4 | 5 | 6 | 7 |  |
| Likely |  |  |  |  |  |  |  | Unlikely |

**I would find the CMOP PROMs app to be flexible to interact with.**

|  | Extremely | Quite | Slightly | Neither | Slightly | Quite | Extremely |  |
| --- | --- | --- | --- | --- | --- | --- | --- | --- |
|  | 1 | 2 | 3 | 4 | 5 | 6 | 7 |  |
| Likely |  |  |  |  |  |  |  | Unlikely |

**It would be easy for me to become skilful at using the CMOP PROMs app.**

|  | Extremely | Quite | Slightly | Neither | Slightly | Quite | Extremely |  |
| --- | --- | --- | --- | --- | --- | --- | --- | --- |
|  | 1 (1) | 2 (2) | 3 (3) | 4 (4) | 5 (5) | 6 (6) | 7 (7) |  |
| Likely |  |  |  |  |  |  |  | Unlikely |

**I would find the CMOP PROMs app easy to use.**

|  | Extremely | Quite | Slightly | Neither | Slightly | Quite | Extremely |  |
| --- | --- | --- | --- | --- | --- | --- | --- | --- |
|  | 1 | 2 | 3 | 4 | 5 | 6 | 7 |  |
| Likely |  |  |  |  |  |  |  | Unlikely |

**How confident are you in the ratings that you have made on this page?**

|  | 1 | 2 | 3 | 4 | 5 | 6 | 7 |  |
| --- | --- | --- | --- | --- | --- | --- | --- | --- |
| Not at all confident |  |  |  |  |  |  |  | Completely confident |

End of Block: PERCIEVED EASE OF USE

Start of Block: PERCEIVED USEFULNESS

**Using CMOP PROMs app to record how my / the person I care for’s treatment affects my / their quality of life would enable me to communicate how my / their treatment impacts quality of life more quickly.**

|  | Extremely | Quite | Slightly | Neither | Slightly | Quite | Extremely |  |
| --- | --- | --- | --- | --- | --- | --- | --- | --- |
|  | 1 | 2 | 3 | 4 | 5 | 6 | 7 |  |
| Likely |  |  |  |  |  |  |  | Unlikely |

**Using CMOP PROMs app would improve how my / the person I care for’s treatment affects my / their quality of life.**

|  | Extremely | Quite | Slightly | Neither | Slightly | Quite | Extremely |  |
| --- | --- | --- | --- | --- | --- | --- | --- | --- |
|  | 1 | 2 | 3 | 4 | 5 | 6 | 7 |  |
| Likely |  |  |  |  |  |  |  | Unlikely |

**Making decisions on treatment with my / the person I care for’s clinician would be more productive if I / we used the CMOP PROMs app.**

|  | Extremely | Quite | Slightly | Neither | Slightly | Quite | Extremely |  |
| --- | --- | --- | --- | --- | --- | --- | --- | --- |
|  | 1 | 2 | 3 | 4 | 5 | 6 | 7 |  |
| Likely |  |  |  |  |  |  |  | Unlikely |

**Using CMOP PROMs app would enhance the effectiveness of the decisions I / we make with my / the person I care for’s clinician on treatment.**

|  | Extremely | Quite | Slightly | Neither | Slightly | Quite | Extremely |  |
| --- | --- | --- | --- | --- | --- | --- | --- | --- |
|  | 1 | 2 | 3 | 4 | 5 | 6 | 7 |  |
| Likely |  |  |  |  |  |  |  | Unlikely |

**Using CMOP PROMs app would make it easier to communicate with my / the person I care for’s clinician how treatment affects my / the person I care for’s quality of life.**

|  | Extremely | Quite | Slightly | Neither | Slightly | Quite | Extremely |  |
| --- | --- | --- | --- | --- | --- | --- | --- | --- |
|  | 1 | 2 | 3 | 4 | 5 | 6 | 7 |  |
| Likely |  |  |  |  |  |  |  | Unlikely |

**I would find CMOP PROMs app useful.**

|  | Extremely | Quite | Slightly | Neither | Slightly | Quite | Extremely |  |
| --- | --- | --- | --- | --- | --- | --- | --- | --- |
|  | 1 | 2 | 3 | 4 | 5 | 6 | 7 |  |
| Likely |  |  |  |  |  |  |  | Unlikely |

**How confident are you in the ratings that you have made on this page?**

|  | 1 | 2 | 3 | 4 | 5 | 6 | 7 |  |
| --- | --- | --- | --- | --- | --- | --- | --- | --- |
| Not at all confident |  |  |  |  |  |  |  | Completely confident |

End of Block: PERCEIVED USEFULNESS

Start of Block: ANTICIPATED USE OF THE CMOP PROMs APP

**For questions on this page, assume the CMOP PROMs app would be available for you to use**.

Imagine you would be able to record how your / the person you care for’s treatment impacts on quality of life, and that you would use it before the clinic appointment. 
The clinician would then be able to see that information, along with other test results, in the clinic appointment.

**Assuming CMOP PROMs app would be available for me / us to use, I predict that I will use it on a regular basis in the future.**

|  | Extremely | Quite | Slightly | Neither | Slightly | Quite | Extremely |  |
| --- | --- | --- | --- | --- | --- | --- | --- | --- |
|  | 1 | 2 | 3 | 4 | 5 | 6 | 7 |  |
| Likely |  |  |  |  |  |  |  | Unlikely |
| Probable |  |  |  |  |  |  |  | Improbable |

**I am most likely to use CMOP PROMs app (pick one):**

- Never
- Rarely
- Occasionally
- Before most of my clinic appointments
- Before every clinic appointment

**I would recommend using the CMOP PROMs app to other cancer patients / carer to record how treatment affects quality of life, so that information can be seen by the clinician in the patient record.**

|  | Extremely | Quite | Slightly | Neither | Slightly | Quite | Extremely |  |
| --- | --- | --- | --- | --- | --- | --- | --- | --- |
|  | 1 | 2 | 3 | 4 | 5 | 6 | 7 |  |
| Likely |  |  |  |  |  |  |  | Unlikely |

**What are the chances in 100 that you will use the CMOP PROMs app to record how your / the person you care for’s treatment impacts your / their quality of life (use the slider)?**

|  | 0 | 10 | 20 | 30 | 40 | 50 | 60 | 70 | 80 | 90 | 100 |  |
| --- | --- | --- | --- | --- | --- | --- | --- | --- | --- | --- | --- | --- |

| Use the slider to indicate () | 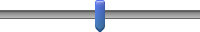 |
| --- | --- |

**How confident are you in the ratings that you have made on this page?**

|  | 1 | 2 | 3 | 4 | 5 | 6 | 7 |  |
| --- | --- | --- | --- | --- | --- | --- | --- | --- |
| Not at all confident |  |  |  |  |  |  |  | Completely confident |

End of Block: ANTICIPATED USE OF THE CMOP PROMs APP

Start of Block: PERCEIVED IMPACT ON CARE

**Assuming I were to use CMOP PROMs app, the quality of the care I / the person I care for receive would be high.**

|  | Extremely | Quite | Slightly | Neither | Slightly | Quite | Extremely |  |
| --- | --- | --- | --- | --- | --- | --- | --- | --- |
|  | 1 | 2 | 3 | 4 | 5 | 6 | 7 |  |
| Likely |  |  |  |  |  |  |  | Unlikely |

**Using CMOP PROMs app, the effectiveness of the care I / the person I care for receive would be:**

|  | Extremely | Quite | Slightly | Neither | Slightly | Quite | Extremely |  |
| --- | --- | --- | --- | --- | --- | --- | --- | --- |
|  | 1 | 2 | 3 | 4 | 5 | 6 | 7 |  |
| LOW |  |  |  |  |  |  |  | HIGH |

**How confident are you in the ratings that you have made on this page?**

|  | 1 | 2 | 3 | 4 | 5 | 6 | 7 |  |
| --- | --- | --- | --- | --- | --- | --- | --- | --- |
| Not at all confident |  |  |  |  |  |  |  | Completely confident |

End of Block: PERCEIVED IMPACT ON CARE

Start of Block: ANTICIPATED ENJOYMENT OF USING THE CMOP PROMs APP

**I would find using the CMOP PROMs app to be enjoyable.**

|  | Extremely | Quite | Slightly | Neither | Slightly | Quite | Extremely |  |
| --- | --- | --- | --- | --- | --- | --- | --- | --- |
|  | 1 | 2 | 3 | 4 | 5 | 6 | 7 |  |
| Likely |  |  |  |  |  |  |  | Unlikely |

**Using the CMOP PROMs app would be:**

|  | Extremely | Quite | Slightly | Neither | Slightly | Quite | Extremely |  |
| --- | --- | --- | --- | --- | --- | --- | --- | --- |
|  | 1 | 2 | 3 | 4 | 5 | 6 | 7 |  |
| Pleasant |  |  |  |  |  |  |  | Unpleasant |

**How confident are you in the ratings that you have made on this page?**

|  | 1 | 2 | 3 | 4 | 5 | 6 | 7 |  |
| --- | --- | --- | --- | --- | --- | --- | --- | --- |
| Not at all confident |  |  |  |  |  |  |  | Completely confident |

End of Block: ANTICIPATED ENJOYMENT OF USING THE CMOP PROMs APP

Start of Block: PATIENT REPORTED OUTCOME MEASURES AND QUALITY OF LIFE

**My / the person I care for’s quality of life, and how treatment impacts it, is:**

|  | Extremely | Quite | Slightly | Neither | Slightly | Quite | Extremely |  |
| --- | --- | --- | --- | --- | --- | --- | --- | --- |
|  | 1 | 2 | 3 | 4 | 5 | 6 | 7 |  |
| Unimportant to me |  |  |  |  |  |  |  | Important to me |
| irrelevant to me |  |  |  |  |  |  |  | Relevant to me |

**I would rate the how difficult I would expect the CMOP PROMs app to use as (use the slider):**

|  | Impossible | Moderate Effort | Effortless |
| --- | --- | --- | --- |

|  |  | 0 | 1 | 2 | 3 | 4 | 5 | 6 | 7 |
| --- | --- | --- | --- | --- | --- | --- | --- | --- | --- |

| Please use the slider () | 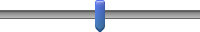 |
| --- | --- |

**I would rate the quality of the CMOP PROMs app as (use the slider):**

|  | Unacceptable (or non-existent) | Moderate Quality | Professional Quality |
| --- | --- | --- | --- |

|  |  | 0 | 1 | 2 | 3 | 4 | 5 | 6 | 7 |
| --- | --- | --- | --- | --- | --- | --- | --- | --- | --- |

| Please use the slider () | 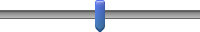 |
| --- | --- |

End of Block: PATIENT REPORTED OUTCOME MEASURES AND QUALITY OF LIFE

Start of Block: OTHER BENEFITS OR CHALLENGES

**We would like to know if you can think of any other benefits or challenges to using the CMOP PROMs app. Please type them in the appropriate boxes below.**

**Benefits of Using the CMOP PROMs Dashboard**

______________________________________________________

**Challenges of Using the CMOP PROMs Dashboard**

______________________________________________________

End of Block: OTHER BENEFITS OR CHALLENGES

[Thank you statement and researcher contact details removed for publication]
