# Supplementary material for: Involving Patients and Clinicians in the Design of Wireframes for Cancer Medicines Electronic Patient Reported Outcome Measures in Clinical Care: Mixed Methods Study
Source: JMIR Form Res. 2023 Dec 21;7:e48296. doi: 10.2196/48296 (PMC10767627; doi:10.2196/48296)
Supplement: Multimedia Appendix 7 [file formative_v7i1e48296_app7.doc]

# Multimedia Appendix 7: Stage 2 Clinician and Patient web-based questionnaire; CHERRIES checklists [1]

This is Multimedia Appendix 7 for a full manuscript published in the JMIR Formative Research. For full copyright and citation information see “Involving Patients and Clinicians in the Design of Wireframes for Cancer Medicines Electronic Patient Reported Outcome Measures in Clinical Care: Mixed Methods Study”.

**Table 1 Clinician Web-Based Questionnaire CHERRIES Checklist**

| **Design** | | |
| --- | --- | --- |
|  | Describe survey design | The target population was clinicians who participated in Stage 1 of the study, plus those recruited via snowball sampling. |
| **IRB (Institutional Review Board) approval and informed consent process** | | |
|  | IRB approval | This study was granted ethical approval by the University of Strathclyde Ethics Committee. |
|  | Informed consent | Informed consent was obtained from all individual participants included in the study. A participant information sheet was included as part of the web-based questionnaire which detailed:   - The purpose of the study - Who the investigator was - That participation was voluntary and they could refuse to participate - Approximately how long the questionnaire would take to complete - What information was being collected, who would have access and how long it would be stored |
|  | Data protection | Participants were told that no identifiable information would be shared about them, only basic demographic information. |
| **Development and pre-testing** | | |
|  | Development and testing | The questionnaire was developed using the TAM model (Davis FD. A technology acceptance model for empirically testing new end-user information systems: theory and results: Massachusetts Institute of Technology; 1986). The questionnaire was reviewed by the project team for face validity. |
| **Recruitment process and description of the sample having access to the questionnaire** | | |
|  | Open survey versus closed survey | The questionnaire was open to allow for snowball sampling |
|  | Contact mode | Initial contact was made via email due to participants having previous involvement in the research programme, either as participants or as part of committees etc. Those recruited via snowball sampling were not initially contacted by the researcher. |
|  | Advertising the survey | Not advertised widely. |
| **Survey administration** | | |
|  | Web/E-mail | Qualtrics© was used to host the survey, and a URL link was provided in emails. |
|  | Context | Qualtrics© is a web-based survey platform, for which the University of Strathclyde has a license. It is not affiliated with any particular domain or area of expertise. |
|  | Mandatory/voluntary | They questionnaire was voluntary. |
|  | Incentives | No incentives were offered. |
|  | Time/Date | Data were collected from October – December 2019. |
|  | Randomisation of items or questionnaires | Items were not randomised. |
|  | Adaptive questioning | No adaptive questioning as all questions were relevant to all participants. |
|  | Number of Items | Total number of items = 40 (including consent) |
|  | Number of screens (pages) | Number of pages = 14 (including Participant Information Sheet, Consent, Instructions and Thank you) |
|  | Completeness check | Participants could not perform completeness check. Questions with a non-mandatory element were:   - Gender - Free text questions asking for any other comments   All questions allowed the selection of one response only. |
|  | Review step | There was no BACK button provided. Participants had the ability to close and restart the questionnaire if they wished. |
| **Response rates** | | |
|  | Unique site visitor | Participants had to provide a name as part of consent process, which was kept confidential, so this could be used to determine if visitors were unique or not. |
|  | View rate (Ratio of unique survey visitors/unique site visitors) | Not calculated |
|  | Participation rate (Ratio of unique visitors who agreed to participate/unique first survey page visitors) | Not calculated |
|  | Completion rate (Ratio of users who finished the survey/users who agreed to participate) | All n=8 participants completed the survey to 100%. |
| **Preventing multiple entries from the same individual** | | |
|  | Cookies used | Not known. |
|  | IP check | IP address is automatically collected via Qualtrics© but participant name provided at consent was used to determine unique visitors. |
|  | Log file analysis | NA. |
|  | Registration | NA. |
| **Analysis** | | |
|  | Handling of incomplete questionnaires | All participants completed the questionnaire to 100%. One participant completed it to 26% but subsequently completed to 100%, and this response was taken into the analysis. |
|  | Questionnaires submitted with an atypical timestamp | NA. |
|  | Statistical correction | NA. |

**Table 2: Patient Web-Based Questionnaire CHERRIES checklist**

| **Design** | | |
| --- | --- | --- |
|  | Describe survey design | The target population was patients attending prostate cancer, gynaecological cancers, and melanoma clinics from two NHS GGC hospitals. Patients were informed about the study by the clinicians who had been involved in Stage 1 through distributing a PIS in-clinic, which contained a URL link to the web-based questionnaire. Some clinicians also provided copies of the patient PIS to colleagues for further distribution while covering their clinics. |
| **IRB (Institutional Review Board) approval and informed consent process** | | |
|  | IRB approval | This study was granted ethical approval by the University of Strathclyde Ethics Committee. |
|  | Informed consent | Informed consent was obtained from all individual participants included in the study. A participant information sheet was included as part of the web-based questionnaire which detailed:   - The purpose of the study - Who the investigator was - That participation was voluntary and they could refuse to participate - Approximately how long the questionnaire would take to complete - What information was being collected, who would have access and how long it would be stored |
|  | Data protection | Participants were told that no identifiable information would be shared about them, only basic demographic information. |
| **Development and pre-testing** | | |
|  | Development and testing | The questionnaire was developed using the TAM model (Davis FD. A technology acceptance model for empirically testing new end-user information systems: theory and results: Massachusetts Institute of Technology; 1986). The questionnaire was reviewed by the project team for face validity. |
| **Recruitment process and description of the sample having access to the questionnaire** | | |
|  | Open survey versus closed survey | The questionnaire was open to allow for snowball sampling |
|  | Contact mode | Patients were recruited by asking clinicians from Stage 1 to distribute copies of a Stage 2 PIS to patients in-clinic, which contained a URL link to the patient (Stage 2) web-based questionnaire. Some clinicians also provided copies of the patient PIS to colleagues for further distribution while covering their clinics. |
|  | Advertising the survey | Not advertised widely. |
| **Survey administration** | | |
|  | Web/E-mail | Qualtrics© was used to host the survey, and a URL link was provided in the PIS. |
|  | Context | Qualtrics© is a web-based survey platform, for which the University of Strathclyde has a license. It is not affiliated with any particular domain or area of expertise. |
|  | Mandatory/voluntary | They questionnaire was voluntary. |
|  | Incentives | No incentives were offered. |
|  | Time/Date | Data were collected from November 2019 to February 2020. |
|  | Randomisation of items or questionnaires | Items were not randomised. |
|  | Adaptive questioning | No adaptive questioning as all questions were relevant to all participants. |
|  | Number of Items | Total number of items = 40 (including consent) |
|  | Number of screens (pages) | Number of pages = 14 (including Participant Information Sheet, Consent, Instructions and Thank you) |
|  | Completeness check | Participants could not perform completeness check. Questions with a non-mandatory element were:   - Free text questions asking for any other comments   All questions allowed the selection of one response only except questions asking what treatments patients were currently or had previously received. |
|  | Review step | A BACK button was provided. |
| **Response rates** | | |
|  | Unique site visitor | Participants had to provide a name as part of consent process, which was kept confidential, so this could be used to determine if visitors were unique or not. |
|  | View rate (Ratio of unique survey visitors/unique site visitors) | Not calculated |
|  | Participation rate (Ratio of unique visitors who agreed to participate/unique first survey page visitors) | Not calculated |
|  | Completion rate (Ratio of users who finished the survey/users who agreed to participate) | Survey metadata showed that n=29 visits were made to the survey (unknown how many were by unique individuals). N=22 unique individuals provided consent and demographics and n=16 (72.7%) went on to complete the survey in full. |
| **Preventing multiple entries from the same individual** | | |
|  | Cookies used | Not known. |
|  | IP check | IP address is automatically collected via Qualtrics© but participant name provided at consent was used to determine unique visitors. |
|  | Log file analysis | NA. |
|  | Registration | NA. |
| **Analysis** | | |
|  | Handling of incomplete questionnaires | N=22 unique individuals provided consent and n=16 (72.7%) went on to complete the survey. All questions in the survey were compulsory apart from free-text responses, therefore there was no missing data.  Any participants who provided consent and demographics but did not complete the questionnaire beyond that were not included in the study. One participant provided consent and demographics and then ceased to complete the survey, but then initiated the survey a second time and completed it in full. |
|  | Questionnaires submitted with an atypical timestamp | NA. |
|  | Statistical correction | NA. |

1. Eysenbach G. Improving the quality of Web surveys: The Checklist for Reporting Results of Internet E-Surveys (CHERRIES). J Med Internet Res. 2004 Sep 29;6(3):e34. doi: 10.2196/jmir.6.3.e34. Erratum in: doi:10.2196/jmir.2042. PMID: 15471760; PMCID: PMC1550605.
