# Supplementary material for: Involving Patients and Clinicians in the Design of Wireframes for Cancer Medicines Electronic Patient Reported Outcome Measures in Clinical Care: Mixed Methods Study
Source: JMIR Form Res. 2023 Dec 21;7:e48296. doi: 10.2196/48296 (PMC10767627; doi:10.2196/48296)
Supplement: Multimedia Appendix 8 [file formative_v7i1e48296_app8.doc]

# Multimedia Appendix 8 Summary of changes to Clinician Dashboard and Patient App from Stage 1.

This is Multimedia Appendix 8 for a full manuscript published in JMIR Formative Research. For full copyright and citation information see “Involving Patients and Clinicians in the Design of Wireframes for Cancer Medicines Electronic Patient Reported Outcome Measures in Clinical Care: Mixed Methods Study”.

**Table 1: Summary of changes to Clinician Dashboard and Patient App from Stage 1.**

|  | **CHANGES MADE** | **CHANGES NOT MADE ^a^** |
| --- | --- | --- |
| **CLINICIAN DASHBOARD** | Detailed *Symptoms and Side Effects* text made larger | Potential for each section to be included in a tab to show more detail over time |
|  | The distress thermometer reduced in size | Consider the currently used chemotherapy booklet and consider adding in to facilitate making any paper-based data collection more integrated |
|  | The pie chart reduced in size |  |
|  | Indicate when reporting of a symptom or side effect is new / there is a change in reporting form last time (e.g. bold text) |  |
|  | Performance status included as a visual / number. |  |
| **PATIENT APP** | Provide both YES and NO options using buttons rather than toggle to accommodate dexterity issues | Link with appointment text alerts and add a PROMs completion reminder. |
|  | Text size made a little bigger overall | The ability to make the app as personalised as possible - suggestions include being able to upload your photo, see case studies of other patients like you, input information on what is important to you etc. |
|  | The *Symptoms & Side Effects* split in two to avoid scrolling |  |
|  | Question numbers should be as limited as is reasonable with as few follow-on questions as possible |  |
|  | Where 0-100 scale used, changed to a 0-10 as much more amenable. |  |
|  | Option to zoom / enlarge pages (likely accommodated for in mobile device’s accessibility settings) | App should also incorporate a feedback system so that patients can view information from the clinician, such as letters, blood test results etc., as well as input information about their quality of life. |
|  | Include NEXT and BACK buttons on all pages |  |
|  | Finger print ID or “keep me signed in” option available |  |
|  | Option for mobile number or CHI number for log in |  |
|  |  | App also available via a webpage |

**^a^** changes that could not be made due to resource constraints, though may be feasible in a final app or working prototype
