# Supplementary material for: Involving Patients and Clinicians in the Design of Wireframes for Cancer Medicines Electronic Patient Reported Outcome Measures in Clinical Care: Mixed Methods Study
Source: JMIR Form Res. 2023 Dec 21;7:e48296. doi: 10.2196/48296 (PMC10767627; doi:10.2196/48296)
Supplement: Multimedia Appendix 9 [file formative_v7i1e48296_app9.doc]

# Multimedia Appendix 9: Stage 2 Clinician (n=8) and Patient (n=16) Median and Interquartile Range (IQRs) Responses to Perceived Ease of Use, Perceived Usefulness and Behavioural Intention to Use Questions

This is Multimedia Appendix 9 for a full manuscript published in JMIR Formative Research. For full copyright and citation information see “Involving Patients and Clinicians in the Design of Wireframes for Cancer Medicines Electronic Patient Reported Outcome Measures in Clinical Care: Mixed Methods Study”.

**Table 1 Stage 2 – Overall Scores for Perceived Ease of Use, Perceived Usefulness and Behavioural Intention to Use ͣ**

|  | **Clinicians (n=8)** | **Patients (n=16)** |
| --- | --- | --- |
|  |  |  |
| **Overall Perceived Ease of Use**  **(median, IQR ᵇ)** | 6 (2 – 6) | 6 (6 – 7) |
| **Overall Perceived Usefulness**  **(median, IQR)** | 4 (3 – 6) | 6 (5 – 6) |
| **Overall Behavioural Intention to Use (median, IQR)** | 6 (4.75 – 6) | 6 (5 – 6) |

ͣ SCALE KEY: Scale used in questionnaire and scores were reversed for data presentation purposes. 1= extremely unlikely, 2= quite unlikely, 3= slightly unlikely, 4=neither likely nor unlikely, 5=slightly likely, 6=quite likely, 7=extremely likely.

*ᵇ IQR = Interquartile Range*

**Table 2 Stage 2 Clinician (n-8)** **Median and Interquartile Range (IQR)) responses to Perceived Ease of Use, Perceived Usefulness and Behavioural Intention to Use Questions ^a^**

|  | **Question** | **Median [IQR]^b^** |
| --- | --- | --- |
| **Perceived Ease of Use** | Learning to operate the CMOP PROMs dashboard would be easy for me. | 6 [2-6] |
|  | I would find it easy to get the CMOP PROMs dashboard to do what I want it to do. | 5.5 [2.75-6] |
|  | My interaction with the CMOP PROMs dashboard would be clear and understandable. | 6 [5.25-6] |
|  | I would find the CMOP PROMs dashboard to be flexible to interact with. | 6 [2.75-6] |
|  | It would be easy for me to become skilful at using the CMOP PROMs dashboard. | 6 [2-6] |
|  | I would find the CMOP PROMs dashboard easy to use. | 5.5 [2-6] |
| **Perceived Usefulness** | Using CMOP PROMs dashboard in my job would enable me to establish the impact treatment has on my patients’ quality of life more quickly. | 5 [2.75-6] |
|  | Using CMOP PROMs dashboard would improve my ability to make decisions on treatment. | 4 [2.75-5.25] |
|  | Using CMOP PROMs dashboard in my job would make my treatment decision making more productive. | 3.5 [2.75-4.5] |
|  | Using CMOP PROMs dashboard would enhance the effectiveness of my treatment decision making. | 3.5 [3-5.25] |
|  | Using CMOP PROMs dashboard would make it easier to do my job. | 3.5 [3-4.5] |
|  | I would find CMOP PROMs dashboard useful in my job. | 4 [3-6] |
| **Behavioural Intention to Use** | Assuming CMOP PROMs dashboard would be available on my job, I predict that I will use it on a regular basis in the future. | 6 [6-6.25] |
|  | I would recommend using the CMOP PROMs dashboard and patient app package to other clinicians to view patient quality of life data, so that information can be used in clinical decision making better. | 5.5 [3.5-6] |

^a^SCALE KEY: Scale used in questionnaire and scores were reversed for data presentation purposes. 1= extremely unlikely, 2= quite unlikely, 3= slightly unlikely, 4=neither likely nor unlikely, 5=slightly likely, 6=quite likely, 7=extremely likely.

*ᵇ IQR = Interquartile Range*

**Table 2 Clinicians (N=8) median and interquartile range (IQR) responses to question on proportion of patients with whom they would use the CMOP dashboard and chance of use**

| **Question** | **Scale key** | **Median (IQR^a^)** |
| --- | --- | --- |
| In my job, I am most likely to use CMOP PROMs dashboard (pick one): | 1= with none of my patients  2= with a small proportion of my patients  3= with about half of my patients  4= with most of my patients  5= with all of my patients | 3.00 [3-4] |
| What are the chances in 100 that you will use the CMOP PROMs dashboard in clinic with patients to enable you to make decisions on their treatment? | Sliding scale, 0-100 | 70 [50-90] |

*^a^IQR = Interquartile Range*

**Table 3 Patient(n=16) Median and Interquartile Range (IQR) responses to Perceived Ease of Use, Perceived Usefulness and Behavioural Intention to Use Questions^a^**

|  | **Question** | **Median [IQR]^b^** |
| --- | --- | --- |
| **Perceived Ease of Use** | Learning to operate the CMOP PROMs app would be easy for me. | 6.5 [6-7] |
|  | I would find it easy to get the CMOP PROMs app to do what I want it to do. | 6 [6-7] |
|  | My interaction with the CMOP PROMs app would be clear and understandable. | 6 [6-7] |
|  | I would find the CMOP PROMs app to be flexible to interact with. | 6 [6-6.25] |
|  | It would be easy for me to become skilful at using the CMOP PROMs app. | 7 [6-7] |
|  | I would find the CMOP PROMs app easy to use. | 6 [6-7] |
| **Perceived Usefulness** | Using the CMOP PROMs app to record how my treatment affects my quality of life would enable me to communicate how my treatment impacts m quality of life more quickly. | 6 [6-6] |
|  | Using CMOP PROMs app would improve how my treatment affects my quality of life. | 5.5 [5-6] |
|  | Making decisions on treatment with my clinicians would be more productive if I used the CMOP PROMs app. | 5.5 [4-6] |
|  | Using CMOP PROMs app would enhance the effectiveness of the decisions I make with my clinician on treatment. | 6 [4.75-6] |
|  | Using CMOP PROMs app would make it easier to communicate with my clinician how treatment affects my quality of life. | 6 [5-6] |
|  | I would find CMOP PROMs app useful. | 6 [5-6] |
| **Behavioural Intention to Use** | Assuming CMOP PROMs app would be available for  me to use, I predict that I will use it on a regular basis in the future. | 6 [5-6] |
|  | I would recommend using the CMOP PROMs app  to other cancer patients to record how treatment affects quality of life, so that information can be seen by the clinician in the patient record. | 6 [3.75-6] |

^a^SCALE KEY: Scale used in questionnaire and scores were reversed for data presentation purposes. 1= extremely unlikely, 2= quite unlikely, 3= slightly unlikely, 4=neither likely nor unlikely, 5=slightly likely, 6=quite likely, 7=extremely likely.

*^b^IQR = Interquartile Range*

**Table 4 Patients (N=16) median and interquartile range (IQR) responses to question on proportion of patients with whom they would use the CMOP dashboard and chance of use**

| Question | Scale key | Median (IQR^a^) |
| --- | --- | --- |
| I am most likely to use CMOP PROMs app (pick one): | 1= Never  2= Rarely  3= Occasionally  4= Before most of my clinic appointments  5= Before every clinic appointment | 4.5 [3.75-5] |
| What are the chances in 100 that you will use the CMOP PROMs app to record how your treatment impacts your quality of life? | Sliding scale, 0-100 | 75 [57.5-80] |

*^a^IQR = Interquartile Range*
